# CD69 mediates the protective role of adipose tissue‐derived mesenchymal stem cells against *Pseudomonas aeruginosa* pulmonary infection

**DOI:** 10.1002/ctm2.563

**Published:** 2021-11-04

**Authors:** Yanshan Jiang, Fan Li, Yanan Li, Jielin Duan, Caixia Di, Yinggang Zhu, Jingya Zhao, Xinming Jia, Jieming Qu

**Affiliations:** ^1^ Department of Respiratory and Critical Care Medicine Ruijin Hospital Affiliated to Shanghai Jiao Tong University School of Medicine Shanghai 200025 China; ^2^ Institute of Respiratory Diseases School of Medicine Shanghai Jiao Tong University Shanghai 200025 China; ^3^ Shanghai Key Laboratory of Emergency Prevention Diagnosis and Treatment of Respiratory Infectious Diseases Shanghai 200025 China; ^4^ Clinical Medicine Scientific and Technical Innovation Center Shanghai Tenth People's Hospital Tongji University School of Medicine Shanghai China; ^5^ Department of Pulmonary and Critical Care Medicine Huadong Hospital Fudan University Shanghai China

**Keywords:** adipose tissue‐derived mesenchymal stem cells, CD69, ERK1, GM‐CSF, *Pseudomonas aeruginosa*

## Abstract

**Background:**

Our previous study shows that Adipose tissue‐derived mesenchymal stem cells (ASCs) are a promising strategy for cell‐based therapy against pulmonary infection with *Pseudomonas aeruginosa* (*P. aeruginosa*), but the underlying mechanisms remain unclear.

**Methods:**

cDNA microarray assay was performed to explore the transcriptome of ASCs primed by *P. aeruginosa*. Small interfering RNA (siRNA) was constructed to select the receptor candidates for *P. aeruginosa* recognition and granulocyte‐macrophage colony‐stimulating factor (GM‐CSF) production in ASCs. The soluble protein chimeras containing the extracellular domain of human CD69 fused to the Fc region of human immunoglobulin IgG1 were used as a probe to validate the recognition of *P. aeruginosa*. The association between CD69 and extracellular regulated protein kinases 1/2 (ERK1/2) was explored via co‐immunoprecipitation, siRNA, and inhibitor. The murine models of *P. aeruginosa* pneumonia treated with WT‐ASCs, *GM‐CSF* ^−/−^‐ASCs *Cd69* ^−/−^‐ASCs or *Erk1* ^−/−^‐ASCs were used to determine the role of GM‐CSF, CD69, and ERK1 in ASCs against *P. aeruginosa* infection.

**Results:**

We showed that C‐type lectin receptor CD69 mediated the protective effects of ASCs partly through GM‐CSF. CD69 could specifically recognize *P. aeruginosa* and regulate GM‐CSF secretion of ASCs. CD69 regulated the production of GM‐CSF via ERK1 in ASCs after *P. aeruginosa* infection. Moreover, the Administration of ASCs with deficiency of CD69 or ERK1 completely blocked its protective effects in a murine model of *P. aeruginosa* pneumonia.

**Conclusions:**

CD69 recognizes *P. aeruginosa* and further facilitates ERK1 activation, which plays a crucial role in ASCs‐based therapy against *P. aeruginosa* pneumonia. CD69 may be a novel target molecule to improve ASCs‐based therapy against *P. aeruginosa* infection.

## Graphical abstract

1. CD69 mediates the protective effects of ASCs partly through GM‐CSF.

2. ASCs use CD69 to recognize *P. aeruginosa* for ERK1 activation and subsequent GM‐CSF production.

3. CD69‐ERK1 pathway plays a key role in ASCs‐based therapy against *P. aeruginosa* pneumonia.

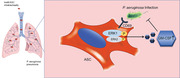

**Abbreviations**
ASCs: adipose tissue‐derived mesenchymal stem cells
ERK: extracellular regulated protein kinases
GM‐CSF: granulocyte‐macrophage colony‐stimulating factor
IL‐1β: interleukin‐1 beta
PCR: polymerase chain reaction
PRR: pattern recognition receptor
TLRs: Toll‐like receptors
TNF‐α: tumor necrosis factor‐alpha

## 1 INTRODUCTION

*Pseudomonas aeruginosa*, a ubiquitous Gram‐negative bacterium, causes life‐threatening pneumonia in immunocompromised and hospitalized patients. With the clinical prevalence of multidrug‐resistant strains, empirical antibiotic therapy is difficult to deal with this microorganism. Therefore, the development of alternative therapeutic strategies for *P. aeruginosa* pneumonia is urgently needed.^1^, ^2^

Adipose tissue‐derived mesenchymal stem cells (ASCs), with the advantages of higher cell concentration and less invasive capability, have more advantages in clinical application than bone‐marrow‐derived mesenchymal stem/stromal cells as well as mesenchymal stem/stromal cells from other origins.^3^ ASCs have been used to protect the host against colitis and sepsis.^4^ Moreover, our previous study revealed that ASCs exhibited protective effects against *P. aeruginosa* pulmonary infection through inhibiting the production of host prostaglandin E2, which subsequently improved phagocytosis and the bactericidal properties of macrophages.^5^ However, the underlying mechanisms for the interaction of ASCs with *P. aeruginosa* remain unclear.

CD69, a marker of lymphocyte activation, is rapidly and transiently induced upon lymphocytes activation and highly expressed on infiltrated leukocytes and endothelial cells.^6^, ^7^, ^8^, ^9^ Recent studies have revealed CD69 as a C‐type lectin receptor with a complex immunoregulatory function other than a regulative marker.^10^, ^11^, ^12^, ^13^, ^14^ The expression and its function of CD69 in ASCs against *P. aeruginosa* infection have not yet been elucidated.

Here, we aimed to clarify the mechanism for ASCs‐mediated protective role against *P. aeruginosa* pneumonia, and explore the function of CD69 expressed on ASCs, which might reveal a novel target molecule to improve ASCs‐based therapy against *P. aeruginosa* infection.

## 2 MATERIALS AND METHODS

### 2.1 Research plan

The study aimed to identify the underlying mechanisms of ASCs‐based therapy against pulmonary infection with *P. aeruginosa*. Briefly, we conducted our protocol based on the following steps. First, to explore the transcriptome of ASCs in response to *P. aeruginosa* infection. Second, to determine whether CD69‐mediated granulocyte‐macrophage colony‐stimulating factor (GM‐CSF) secreted by ASCs alleviated *P. aeruginosa* infection with Quantitative real‐time polymerase chain reaction (PCR), ELISA, recombinant protein, as well as knocking out the expression of receptor candidates by CRISPR/Cas9 system. Last, to investigate the role of CD69‐ERK1 pathway in ASCs‐based therapy against *P. aeruginosa* pneumonia via co‐immunoprecipitation, siRNA, and inhibitor.

In all in vivo experiments, mice were assigned randomly and at least three mice were used for each group. No blinding approach was adopted. At least three independent experimental replicates were performed. Analytical studies were implementing fixed time points of analysis for all experimental groups.

### 2.2 Study animals and subjects

#### 2.2.1 Mice

The sources and identifiers of the mice used in this article can be found in Table S2. The genotype of GM‐CSF‐deficient (*GM‐CSF* ^−/−^) mice was identified by PCR (Figure S6A) and the knockout efficiency of GM‐CSF of ASCs was confirmed by ELISA (Figure S6B). We made *Cd69* knockout mice and *Erk1* knockout mice via the CRISPR/Cas9 system. *ERK2*‐null results in the death of mice early during embryonic development,^15^ so we did not make *Erk2* ^−/−^‐deficient mice. The detailed information can be found in Supporting Information.*Cd69* ^−/−^ mice and *Erk1* ^−/−^ mice were backcrossed for more than eight generations to the C57BL/6J background. The genotype of *Erk1* ^−/−^ mice was confirmed by PCR (Figure S6C). Western blot was used to confirm ERK1 knockout efficiency in ASCs (Figure S6D). The genotype of *Cd69* ^−/−^ mice was confirmed by PCR (Figure S6E). Western blot was used to confirm the CD69 knockout efficiency of mice (Figure S6F). Quantitative real‐time PCR was used to confirm the CD69 knockout efficiency of ASCs (Figure S6G). All mice used in this study were on the C57BL/6 background. All mice were housed in the specified‐pathogen‐free facilities in the Research Center for Experimental Medicine of Ruijin Hospital affiliated to Shanghai Jiao Tong University School of Medicine or at Tongji University. Mice experiments were performed only on male mice in all conditions.

#### 2.2.2 Bacterial culture

The *P. aeruginosa* WT strain, PAO1, GFP‐PAO1 were used in the experiment according to our previous study.^5^ In brief, 200 μl bacteria frozen stock were grown in 5 ml of sterile Luria–Bertani medium for 18 h at 37°C and stirred at 200 rpm. Before use, the bacterial cells were washed twice and resuspended in phosphate buffered saline (PBS) for optical density (OD at k ¼ 600 nm), and then diluted to the suitable concentration.

#### 2.2.3 ASCs isolation

ASCs were isolated and purified as in our previous study.^5^ Briefly, adipose tissue, harvested from the inguinal area of 8 weeks old male mice, was digested with 0.1% (wt/vol) collagenase type I (Sigma‐Aldrich, USA) in 37°C stirred at 200 rpm for 50 min. ASCs were cultured in the mixed medium of Dulbecco's modified Eagle's medium and Nutrient Mixture F‐12 (DMEM/F12; Gibco, Thermo Fisher Scientific, USA) supplemented with 10% (vol/vol) fetal bovine serum (Gibco; Thermo Fisher Scientific, USA) and 1% (vol/vol) penicillin/streptomycin (Gibco; Thermo Fisher Scientific, USA), and cultured on flasks at 37°C with 5% CO_2_. When the confluence reached 80%–90% confluent, and the cells were digested for passage. Cells of the fourth generation were used in our study.

#### 2.2.4 Acute *P. aeruginosa* pneumonia mice and ASCs treatment model

Mice (7–8 weeks old, male) were anesthetized with pentobarbital sodium salt (20 μg/g, Merk; Merck KGaA, Darmstadt, Germany, #1063180500) by intraperitoneal administration. Note that 40 μl PBS with PAO1 (3 × 10^6^) was delivered using a sterile 24‐gauge SURFLOVR Flash I.V. Catheter (TERUMO, Japan). Four hours post‐PAO1 infection, mice were treated intratracheally with 1 × 10^6^ ASCs in 40 μl PBS by the same method.

Twenty‐four hours post‐PAO1 infection, the lungs of mice were homogenized for bacterial burden assessment or fixed with 4% (vol/vol) paraformaldehyde for histopathological analysis.

### 2.3 Methods

#### 2.3.1 Enrichment of ASCs after transplant by fluorescent‐activated cell sorting

Post‐PAO1 infection, mice were treated intratracheally with GFP‐ASCs coming from GFP expressing mice as the method above. Mice were killed 8 h after the instillation of PAO1. The lungs were digested into single‐cell suspensions as reported previously.^16^ After lysing the erythrocytes, the single‐cell suspensions were sorted for GFP‐ASCs using Aria II cell sorter (BD Biosciences). The total RNA of sorted GFP‐ASCs was extracted with the RNeasy Micro Kit (Qiagen) for Quantitative real‐time PCR reaction analysis.

#### 2.3.2 Expression and purification of protein chimeras containing the extracellular domain of human CD69 fused to the Fc region of human immunoglobulin IgG1

We made the hCD69‐hFc fusion proteins according to previous reports.^17^ Briefly, the cDNA encoding the extracellular parts of hCD69 (CCDS 8604.1) was applied by PCR using specific primers (Table S1), and then ligated into a pFuse‐hIgG1‐Fc expression vector (InvivoGen, USA). The wild‐type plasmid which including all the extracellular parts of hCD69 is hCD69‐pFuse‐hIgG1‐Fc. Next, 293T cells were transiently transfected with the vectors using Lipofectamine 3000 (Thermo Fisher Scientific, USA) according to the manufacturer's protocol. After 6 h of transfection, the medium was replaced with DMEM (Gibco; Thermo Fisher Scientific, USA) without fetal bovine serum or penicillin/streptomycin. Cell supernatant was purified after 3 days of transfection using dialysis membranes (7000D; Solarbio). Then purified cell supernatant was concentrated 10 times by polyethylene glycol‐2000 (PEG 2000; Sangon Biotech). To confirm the purity of hCD69‐hFc, proteins in this supernatant were analyzed by western blot using an anti‐hFc antibody (Jackson ImmunoResearch).

#### 2.3.3 Mutant preparation of hCD69‐hFc recombinant proteins

hCD69‐hFc mutagenesis plasmid was performed using Fast Site‐Directed Mutagenesis Kit (TIANGEN) with hCD69‐pFuse‐hIgG1‐Fc as the template according to the manufacturer's protocols. All the mutants were prepared in the same methodical manner as the wild type. The primers can be found in Table S1.

#### 2.3.4 Binding assays

The analyses of hCD69‐hFc binding were performed as previously reported.^17^, ^18^ Briefly, 1 × 10^7^ CFU PAO1 were incubated with purified hCD69‐hFc recombinant proteins for 1 h at 37°C. After washing, the bacterial pellet was stained with FITC‐conjugated anti‐hFc antibody (Jackson ImmunoResearch) for 30 min at 4°C. Flow‐cytometric analysis was performed as in Supporting Information. hFc protein was used as the control to exclude the nonspecific binding of PAO1 to the Fc part of the hCD69‐hFc.

We also used the confocal technique to further verify the binding of *P. aeruginosa* with hCD69‐hFc. GFP‐PAO1 was prepared following the above method and stained with Alexa Fluor 647‐conjugated anti‐hFc antibody (Jackson ImmunoResearch). GFP‐PAO1 was analyzed with Nikon A1R.

#### 2.3.5 siRNA transfection

Small interfering RNAs (siRNAs) and negative control (NC) were purchased from Genepharma (Shanghai, China). Sequences used in this study are in Table S1. Lipofectamine RNAiMAX (Thermo Fisher Scientific, USA) was used to transfect ASCs with siRNA, following the manufacturer's instructions. ASCs were harvested for analysis of the knockdown efficiency by western blot 72 h after transfection.

#### 2.3.6 Microarray analysis

Total RNA in ASCs infected with PAO1 for 0 and 4 h was extracted using TRIzol (Thermo Fisher Scientific). The sample labeling, microarray hybridization, and washing were performed based on the manufacturer's standard protocols. The hybridization signals were detected by the Agilent Scanner G2505C (Agilent Technologies). Genespring (version13.1, Agilent Technologies) was employed to finish the basic analysis. The probes that at least 100% of the values in one out of all conditions have flags in "Detected" were chosen for further data analysis. The threshold set for up‐ and down‐regulated genes was a fold change ≥2.0 and a *p*‐value ≤ 0.05. Afterward, GO analysis and heat map analysis was performed on the differentially expressed genes.

#### 2.3.7 Co‐immunoprecipitation assay

ASCs were stimulated with or without PAO1, and the cells were collected with Cell lysis buffer for Western and IP (Beyotime, Shanghai, China) supplemented with phenylmethanesulfonyl fluoride. The cell lysates were immunoprecipitated with the indicated antibody‐conjugated Protein A/G‐Agarose (Abmart). Other methods can be found in the Supporting Information.

#### 2.3.8 Ethics statement

Animal studies were approved by the Ethics Committee of Ruijin Hospital affiliated to Shanghai Jiao‐Tong University School of Medicine.

#### 2.3.9 Statistical analyses

Prism 8.0 software (GraphPad) was used for statistical analysis and graphs formation. Statistical analyses were performed using one‐way ANOVA with *Tukey's* multiple comparisons test or two‐tailed unpaired *t*‐test as indicated in figure legends. *p‐*values below 0.05 were considered significant. The number of independent biological replicates is indicated as *n* in the figure legends. Results are presented as mean ± SEM and are representative of three independent experiments: ^*^ *p *< 0.05, ^**^ *p *< 0.01, ^***^ *p *< 0.005, ^****^ *p *< 0.001; *NS*, not significant. Flow cytometric analysis data were analyzed using Flow Jo software V10 (Tree Star).

## 3 RESULTS

### 3.1 GM‐CSF secreted by ASCs exhibits the protective role against *P. aeruginosa* pneumonia

**FIGURE 1:**
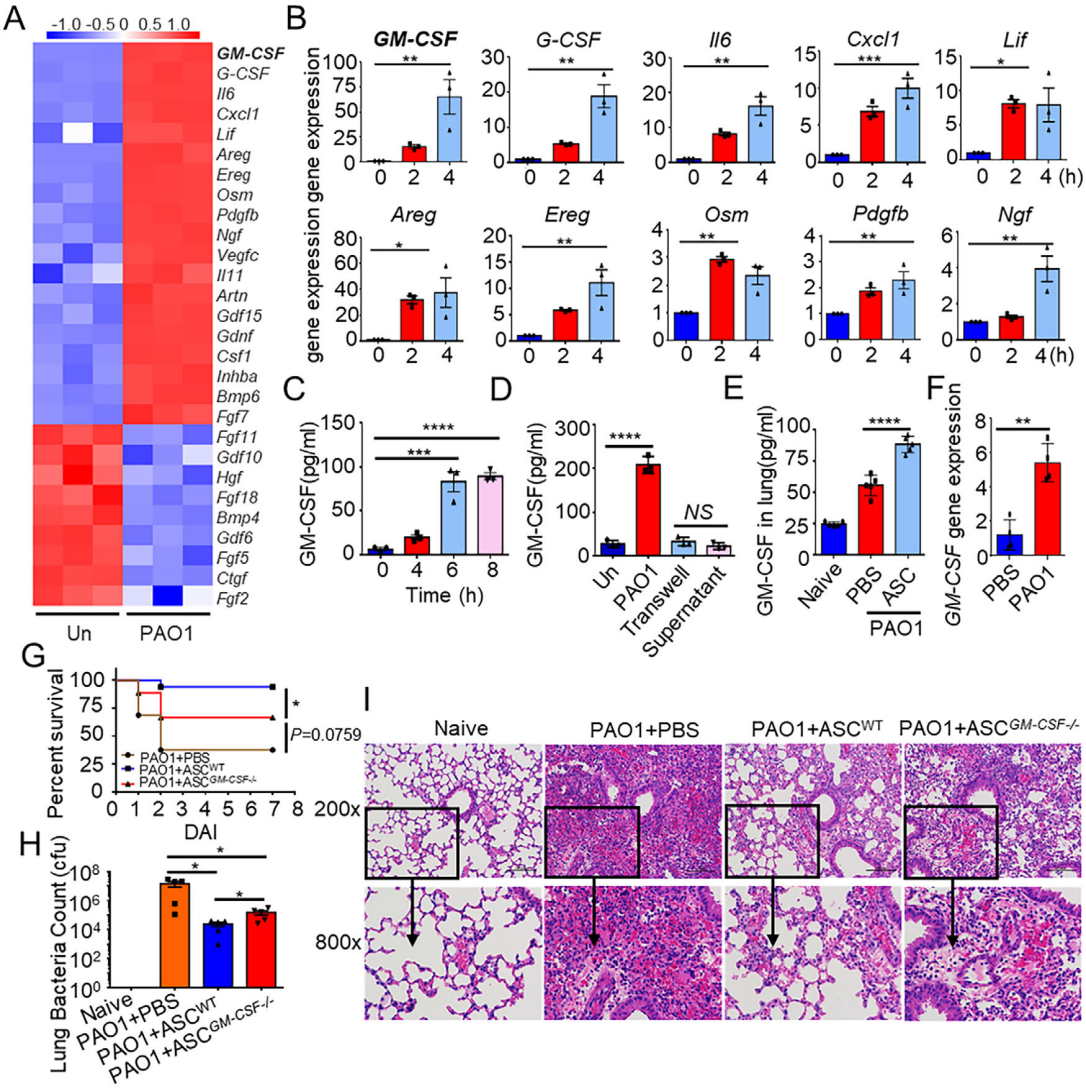
Granulocyte‐macrophage colony‐stimulating factor (GM‐CSF) secreted by adipose tissue‐derived mesenchymal stem cells (ASCs) exhibits the protective role against *Pseudomonas aeruginosa* (*P. aeruginosa*) pneumonia. (A) Heat map of microarray analysis data showing differentially expressed growth factor genes (right margin) in ASCs after stimulation with PBS (Un) or PAO1 for 4 h (*n* = 3/group) (below lanes). (B) Quantitative real‐time polymerase chain reaction (PCR) analysis of top 10 growth factor genes in ASCs stimulated with PAO1 (MOI = 1) for 0, 2, or 4 h; results were normalized to those of the control gene *β‐actin* (*n* = 3/group). (C) ELISA results for GM‐CSF in supernatants of ASCs stimulated with PAO1 (MOI = 1) for 0, 4, 6, or 8 h (*n* = 3/group). (D) ELISA results for GM‐CSF in supernatants of ASCs stimulated with PAO1 by co‐incubation or by transwell, or the supernatants that cultivated PAO1 for 8 h and were filtrated out PAO1 with 0.22 um filter unit, at MOI = 1 for 8 h (*n* = 3/group). (E) Mice were infected with 3 × 10^6^ CFU PAO1 by i.t., then given therapeutic delivery of PBS or 1 × 10^6^ WT‐ASCs by i.t. 4 h later. ELISA results for GM‐CSF in lung tissue lysate supernatants of mice treated with WT‐ASCs or PBS at 24 h post‐infection (hpi; *n* = 5/group). (F) Quantitative real‐time PCR analysis of *GM‐CSF* expression in enriched GFP‐ASCs from the lungs of WT mice treated with PAO1 or PBS (n = 4/group). (G–I) Mice were infected with 3 × 10^6^ CFU PAO1 by i.t., then given therapeutic delivery of PBS, 1 × 10^6^ WT‐ASCs, or *GM‐CSF* ^−/−^‐ASCs by i.t. 4 h later, respectively. (G) Survival of mice treated with PBS (*n* = 16), WT‐ASCs (*n* = 17), or *GM‐CSF* ^−/−^‐ASCs (*n* = 18) for 7 days. (H) CFU assay of lung tissue homogenate at 24 hpi (*n* = 5/group). (I) Representative pathological sections of lungs were analyzed with hematoxylin and eosin (H&E) staining at 24 hpi. Statistical difference calculated by one‐way ANOVA with Tukey's multiple comparisons test (B–E, H), two‐tailed unpaired *t*‐test (F), or log‐rank (Mantel–Cox) test (G). Results are presented as mean ± SEM and are representative of three independent experiments. ^*^ *p *< 0.05, ^**^ *p *< 0.01, ^***^ *p *< 0.005, ^****^ *p *< 0.001; *NS*, not significant

To explore the mechanism of ASCs exerting protection against *P. aeruginosa* infection, we performed a cDNA microarray assay of ASCs primed by *P. aeruginosa* or PBS. Gene Ontology analyses suggested that *P. aeruginosa* stimulation induced a significant change of various growth factor genes (Figure S1A). Heat map analysis showed that *GM‐CSF* was the most significantly upregulated (Figure 1A) after *P. aeruginosa* stimulation, with about 60 folds increment, which was verified by Quantitative real‐time PCR (Figure 1B). Consistently, the ELISA assay confirmed increased production of GM‐CSF in ASCs stimulated with *P. aeruginosa* (Figure 1C). However, GM‐CSF production was undetected when ASCs were co‐cultured with *P. aeruginosa* using a transwell system or treated with the supernatant of *P. aeruginosa* culture media (Figure 1D). These findings revealed that *P. aeruginosa* itself, but not its metabolites, directly induced GM‐CSF secretion of ASCs.

To determine whether *P. aeruginosa* infection could induce *GM‐CSF* expression of ASCs in vivo, we intratracheally instilled PBS or ASCs into *P. aeruginosa* pneumonia mice and found that ASCs treated group presented a higher level of GM‐CSF in the lung when compared with the PBS control group (Figure S1B and Figure 1E). Furthermore, we transplanted intratracheally GFP‐expressing ASCs (GFP‐ASCs) into the mice with or without *P. aeruginosa* infection. We sorted GFP‐ASCs from the lung via fluorescent‐activated cell sorting (Figure S1C) and found that the expression of *GM‐CSF* was significantly increased in GFP‐expressing ASCs (Figure 1F). These findings revealed that *P. aeruginosa* infection could increase the GM‐CSF production of ASCs in vivo.

To investigate whether GM‐CSF from ASCs could mediate the protective effects against *P. aeruginosa* pneumonia, we intratracheally instilled PBS, *GM‐CSF* ^−/−^‐ASCs, or WT‐ASCs into *P. aeruginosa* pneumonia mice. Consistently with previous reports, administration with WT‐ASCs dramatically decreased mortality (Figure 1G), pulmonary bacterial burden (Figure 1H), and the severity of lung injury (Figure 1I) compared with those from the PBS treatment group. However, administration of *GM‐CSF* ^−/−^‐ASCs significantly increased mortality (Figure 1G), pulmonary bacterial burden (Figure 1H), and the severity of lung injury (Figure 1I) compared with those from the WT‐ASCs group. Together, these findings indicated that GM‐CSF secreted by ASCs could at least partially alleviate *P. aeruginosa* pneumonia.

### 3.2 GM‐CSF secretion is independent of TLR2, TLR4, NLRP3, or NLRC4

**FIGURE 2:**
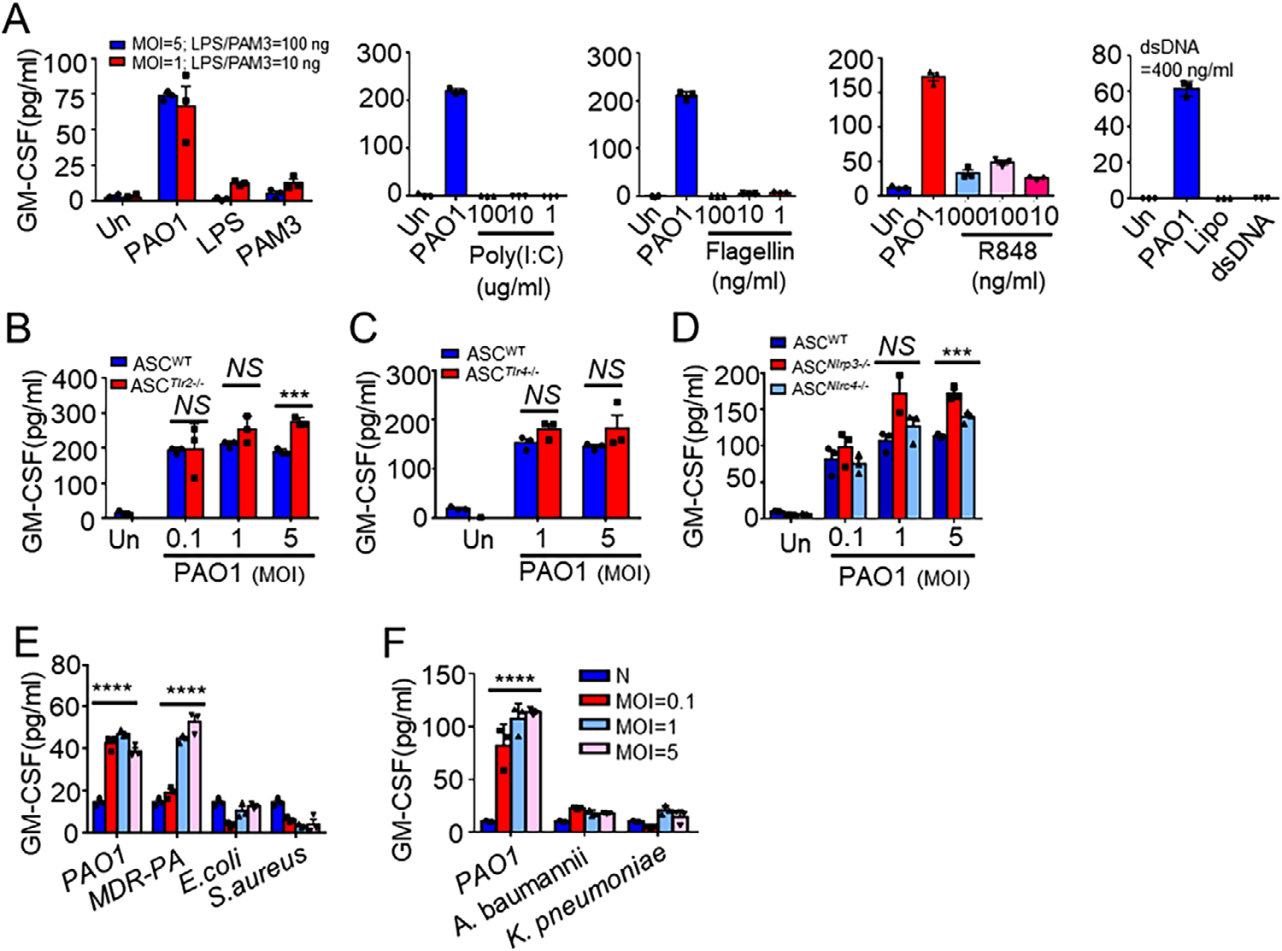
Granulocyte‐macrophage colony‐stimulating factor (GM‐CSF) secretion is independent of TLR2, TLR4, NLRP3, or NLRC4. (A) ELISA results for GM‐CSF in supernatants of adipose tissue‐derived mesenchymal stem cells (ASCs) treated with PAO1 at MOI = 1, 5 or various agonists of Toll‐like receptors (TLRs) at indicated concentrations for 8 h (*n* = 3/group). LPS (Lipopolysaccharide, TLR4 agonist); PAM3 (pam3CSK4, TLR1/TLR2 agonist); Poly(I:C) (polyinosinic‐polycytidylic acid, TLR3 agonist); Flagellin (TLR5 agonist); R848 (TLR7/8 agonist); dsDNA (TLR9 agonist). (B, C) ELISA results for GM‐CSF in supernatants of WT‐ASCs, *Tlr2* ^−/−^‐ASCs or *Tlr4* ^−/−^‐ASCs stimulated with PAO1 at various MOIs as indicated for 8 h (*n* = 3/group). (D) ELISA results for GM‐CSF in supernatants of WT‐ASCs, *Nlrp3* ^−/−^‐ASCs or *Nlrc4* ^−/−^‐ASCs stimulated with PAO1 at various MOIs as indicated for 8 h (*n* = 3/group). (E, F) ELISA detection for GM‐CSF in supernatants of ASCs stimulated with indicated bacteria (MOI = 0.1, 1, and 5) for 8 h (*n* = 3/group). Statistical difference calculated by one‐way ANOVA with Tukey's multiple comparisons test (D–F), or two‐tailed unpaired *t*‐test (B, C). Results are presented as mean ± SEM and are representative of three independent experiments. ^***^ *p *< 0.005, ^****^ *p *< 0.001; *NS*, not significant

Accumulating evidence has demonstrated that Toll‐like receptors (TLRs) signaling is pivotal for GM‐CSF secretion.^19^, ^20^ To test whether GM‐CSF production in ASCs was dependent on TLRs signaling, we stimulated ASCs with various TLRs agonists. We found that TLRs agonists failed to induce GM‐CSF secretion of ASCs (Figure 2A). Meanwhile, we stimulated ASCs isolated from *Tlr2* ^−/−^, *Tlr4* ^−/−^, and WT mice with *P. aeruginosa*, and found that GM‐CSF production was independent of these two TLRs (Figure 2B,C). Moreover, we also assessed GM‐CSF production by ASCs isolated from *Nlrp3* ^−/−^, *Nlrc4* ^−/−^ and WT mice, and found that deficiency of *Nlrp3* and *Nlrc4* exhibited no effects on GM‐CSF secretion in ASCs stimulated with *P. aeruginosa* (Figure 2D). Notably, a significant secretion of GM‐CSF was confirmed when ASCs stimulated with multidrug‐resistant *P. aeruginosa*, but not with other pathogens, such as *Escherichia coli*, *Staphylococcus aureus*, *Acinetobacter baumannii*, and *Klebsiella pneumoniae* (Figure 2E,F). The corresponding number of survival ASCs was counted at different MOI levels (Figure S2A,B). These data suggested a novel pattern recognition receptor (PRR) for *P. aeruginosa* recognition and GM‐CSF production in ASCs.

### 3.3 CD69 mediates GM‐CSF and some inflammatory cytokines secretion of ASCs in response to *P. aeruginosa* challenge

**FIGURE 3:**
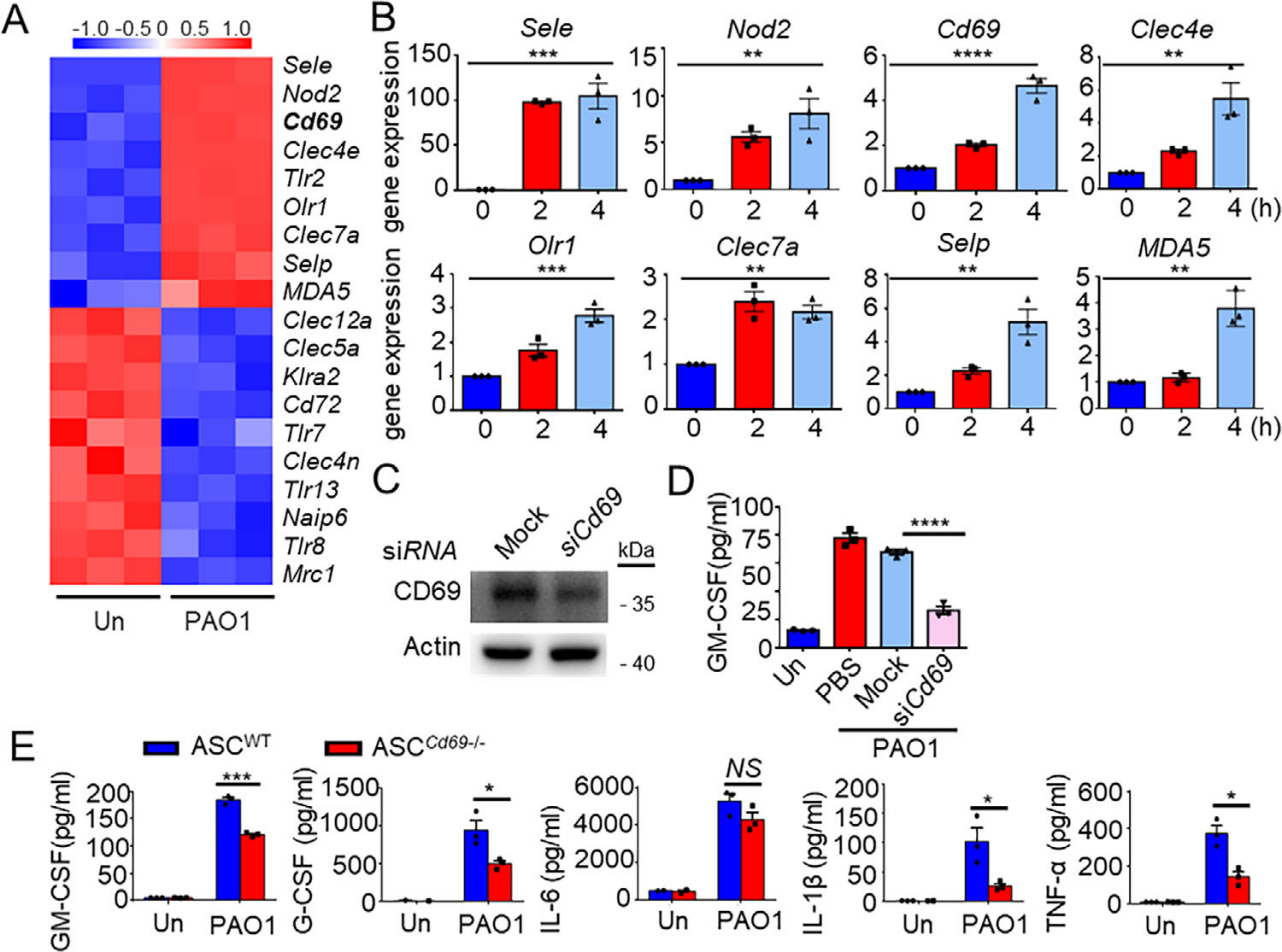
CD69 mediates granulocyte‐macrophage colony‐stimulating factor (GM‐CSF) and some inflammatory cytokines secretion of adipose tissue‐derived mesenchymal stem cells (ASCs) in response to *Pseudomonas aeruginosa* (*P. aeruginosa*) challenge. (A) Heat map of microarray analysis data showed differentially expressed pattern recognition receptors (PRRs) genes (right margin) in ASCs after stimulation with PBS (Un) or PAO1 (*n* = 3/group) (below lanes). (B) Quantitative real‐time polymerase chain reaction (PCR) analysis of PRRs expression in ASCs stimulated with PAO1 (MOI = 1) for 0, 2, or 4 h; results were normalized to those of the control gene *β‐actin* (*n* = 3/group). (C, D) ASCs were transfected with small interfering RNA (siRNA) for CD69 or non‐target dsRNA (Mock). (C) Relative protein expression of CD69 and GAPDH (loading control) was determined by western blot analysis; (D) GM‐CSF in supernatants of these ASCs stimulated with PAO1 (MOI = 1) for 8 h was determined by ELISA (*n* = 3/group). (E) ELISA results for GM‐CSF, G‐CSF, IL‐6, IL‐1β, and TNF‐α in supernatants of WT‐ASCs and *Cd69* ^−/−^‐ASCs stimulated with PAO1 at MOI = 1 for 8 h (*n* = 3/group). Statistical difference calculated by one‐way ANOVA with *Tukey's* multiple comparisons test (B, D), or two‐tailed unpaired *t*‐test (E). Results are presented as mean ± SEM and are representative of three independent experiments.^*^ *p *< 0.05, ^**^ *p *< 0.01, ^***^ *p *< 0.005, ^****^ *p *< 0.001; *NS*, not significant

To identify the potential receptors recognizing *P. aeruginosa*, we clustered all PRRs with different levels of expression between *P. aeruginosa*‐primed and PBS‐primed ASCs using cDNA microarray assay. We screened 8 genes of PRRs which exhibited higher expression in *P. aeruginosa*‐stimulated ASCs (Figure 3A), which were subsequently verified by Quantitative real‐time PCR (Figure 3B). To determine which PRR was crucial for GM‐CSF production in ASCs, we stimulated ASCs isolated from *Clec7a* ^−/−^, *Clec4e* ^−/−^, and WT mice with *P. aeruginosa*. We found that deficiency of *Clec7a* and *Clec4e* had no effects on GM‐CSF secretion in ASCs in response to *P. aeruginosa* stimulation (Figure S3A). Furthermore, knocking down the expression of *Olr1* or *MDA5* in ASCs by siRNA also had no impact on GM‐CSF production in ASCs in response to *P. aeruginosa* stimulation (Figure S3B‐E). However, the GM‐CSF production was dramatically decreased (dropped by 61%) while knocking down the expression of *Cd69* (Figure 3C,D).

Consistently, deficiency of CD69 significantly reduced GM‐CSF production by 34.52% in ASCs after *P. aeruginosa* stimulation. Interestingly, CD69 deficiency also significantly reduced the secretion of G‐CSF (granulocyte‐colony stimulating factor) by 47.18%, IL‐1β (interleukin‐1 beta) by 74.64%, and TNF‐α (tumor necrosis factor‐alpha) by 61.55%, while CD69 deficiency had no effect on the production of IL‐6 (interleukin‐6) in ASCs after *P. aeruginosa* stimulation (Figure 3E). These data suggested that CD69 might be a receptor for *P. aeruginosa* recognition and GM‐CSF as well as other inflammatory cytokines production in ASCs.

### 3.4 CD69 can specifically recognize *P. aeruginosa*

**FIGURE 4:**
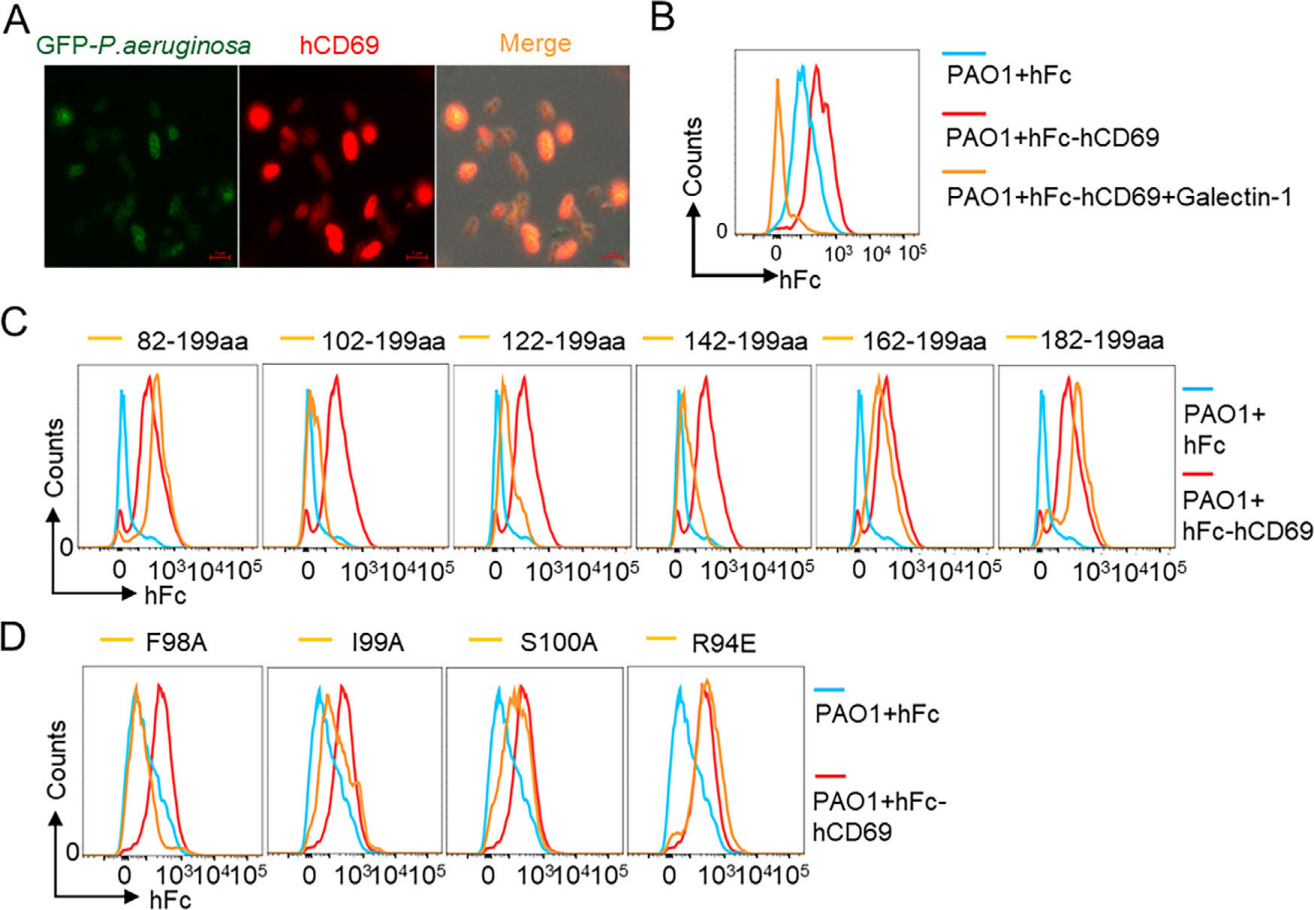
CD69 can specifically recognize *Pseudomonas aeruginosa* (*P. aeruginosa*). (A) Representative confocal images of GFP‐PAO1(green) stained with hCD69‐hFc (red). Scale bar = 2 μm. (B) Binding analysis of soluble protein chimera hCD69‐hFc to PAO1 with or without Galectin‐1 by flow cytometry using hFc as a control. (C) Flow cytometry analysis of binding of PAO1 with indicated soluble protein truncations of hCD69‐hFc. (D) Flow cytometry was used to analyze the binding of the different point‐mutated fusion proteins of hCD69‐hFc to PAO1. Results are representative of three independent experiments

To determine whether CD69 was a receptor for *P. aeruginosa* recognition, we generated soluble protein chimeras containing the extracellular domain of human CD69, including 62–199 amino acids residues, fused to the Fc region of human immunoglobulin IgG1 (hCD69‐hFc), and used hCD69‐hFc as a probe to validate the recognition of *P. aeruginosa*. By confocal microscopy assay, which revealed a uniform overlap of hCD69 with *P. aeruginosa*, we identified that hCD69 bound with *P. aeruginosa in vitro* (Figure 4A). Flow cytometry assay further confirmed the specific binding of hCD69 with *P. aeruginosa*, and the binding of hCD69 with *P. aeruginosa* could be competitively inhibited by soluble recombinant Gal‐1, one of the putative CD69 ligands^18^, ^21^ (Figure 4B).

Next, we generated soluble protein chimeras containing different extracellular fragments of hCD69 to identify the exact binding site of hCD69 to *P. aeruginosa* (Figure S4A). Flow cytometry analyses showed that the truncation containing 82–199 amino acids residues could bind with *P. aeruginosa*, while the truncation containing 102–199 amino acids residues completely lost the binding with *P. aeruginosa*, suggesting 82–101 amino acids residues in hCD69 might be critical for their binding. However, the truncation containing 182–199 amino acids residues could also bind with *P. aeruginosa* (Figure S4B and Figure 4C). These data indicated that the extracellular domain of hCD69 contained at least two binding sites with *P. aeruginosa*.

We subsequently generated soluble protein chimeras containing mutations of extracellular fragments of hCD69, respectively substituting Met^184^, Leu^190^, Trp^192^, Ile^193^, Phe^98^, Ile^99^, Ser^100^ with Alanine or Met^184^, Lys^196^, Lys^199^, Lys^188^ Arg^94^ with Glutamic acid to clarify the *P. aeruginosa* binding site on hCD69 (Figure S4C–E and Figure 4D). We found mutations at Phe^98^ and Ile^99^ completely impaired the binding of hCD69 with *P. aeruginosa*. These data indicated that Phe^98^ and Ile^99^ were crucial for hCD69 binding with *P. aeruginosa* and confirmed the specific recognition of *P. aeruginosa* by hCD69.

### 3.5 CD69 deficiency completely blocks the protective role of ASCs against *P. aeruginosa* pneumonia

**FIGURE 5:**
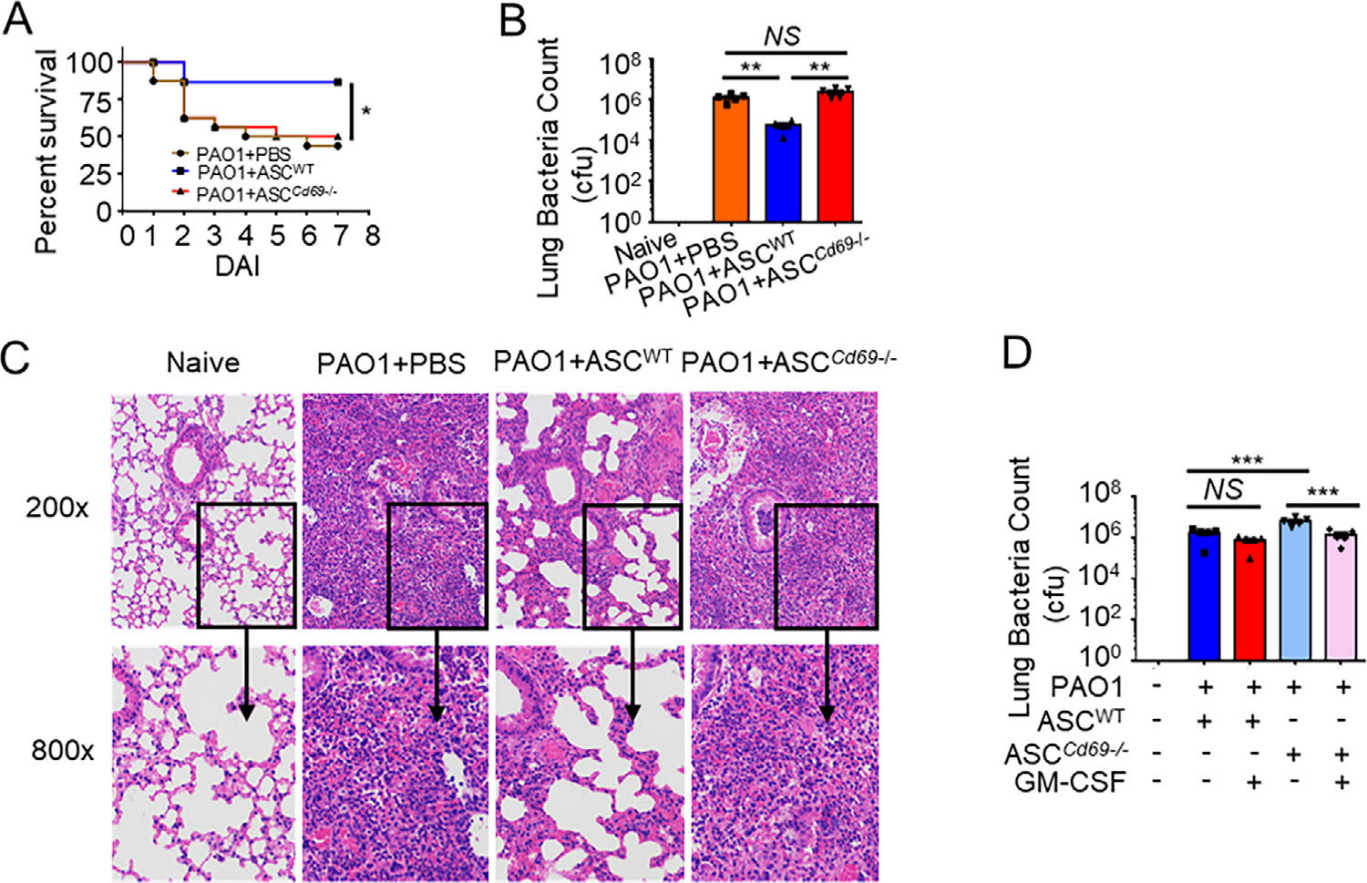
CD69 deficiency completely blocks the protective role of ASCs against *Pseudomonas aeruginosa* (*P. aeruginosa*) pneumonia. (A–D) Mice were infected with 3 × 10^6^ CFU PAO1 by i.t., then given therapeutic delivery of PBS, 1 × 10^6^ WT‐ASCs, 1 × 10^6^ WT‐ASCs with granulocyte‐macrophage colony‐stimulating factor (GM‐CSF), 1 × 10^6^ *Cd69* ^−/−^‐ASCs or 1 × 10^6^ *Cd69* ^−/−^‐ASCs with GM‐CSF (100 ng/mouse) by i.t. 4 h later, respectively. (A) Survival of mice receiving PBS (*n* = 16), WT‐ASCs (*n* = 15), or *Cd69* ^−/−^‐ASCs (*n* = 16) for 7 days. (B) CFU assay of lung tissue homogenate from mice receiving PBS, WT‐ASCs, or *Cd69* ^−/−^‐ASCs at 24 hpi (*n* = 5/group). (C) Representative pathological sections of lungs from mice receiving PBS, WT‐ASCs, or *Cd69* ^−/−^‐ASCs analyzed with hematoxylin and eosin (H&E) staining at 24 hpi. (D) CFU assay of lung tissue homogenate from mice receiving WT‐ASCs, WT‐ASCs with GM‐CSF, *Cd69* ^−/−^‐ASCs, or *Cd69* ^−/−^‐ASCs with GM‐CSF at 24 hpi (*n* = 5/group). Statistical difference calculated by one‐way ANOVA with *Tukey's* multiple comparisons test (B, D), or *log‐rank* (*Mantel‐Cox*) test (A). Results are presented as mean ± SEM and are representative of three independent experiments. ^*^ *p *< 0.05, ^**^ *p *< 0.01, ^***^ *p *< 0.005; *NS*, not significant

To investigate the effects of CD69 on ASCs against *P. aeruginosa* pneumonia, PBS, WT‐ASCs, or *Cd69* ^−/−^‐ASCs were transplanted into *P. aeruginosa* pneumonia mice. Administration of *Cd69* ^−/−^‐ASCs presented higher mortality (Figure 5A), bacterial burden (Figure 5B), and severity of lung injury (Figure 5C) compared with the WT‐ASCs treated group, while there were no significant differences between the PBS control group and *Cd69^−/−^*‐ASCs treated group. Moreover, administration of GM‐CSF with *Cd69^−/−^*‐ASCs could increase bacteria clearance effects of *Cd69^−/−^*‐ASCs (Figure 5D), while administration of GM‐CSF with WT‐ASCs did not affect the bacteria clearance effects of WT‐ASCs. These data indicated that CD69 was critical for ASCs against *P. aeruginosa* infection, and GM‐CSF was involved in the protective effects of ASCs mediated by CD69.

### 3.6 ERK1/2 are critical regulators of GM‐CSF secretion in ASCs

**FIGURE 6:**
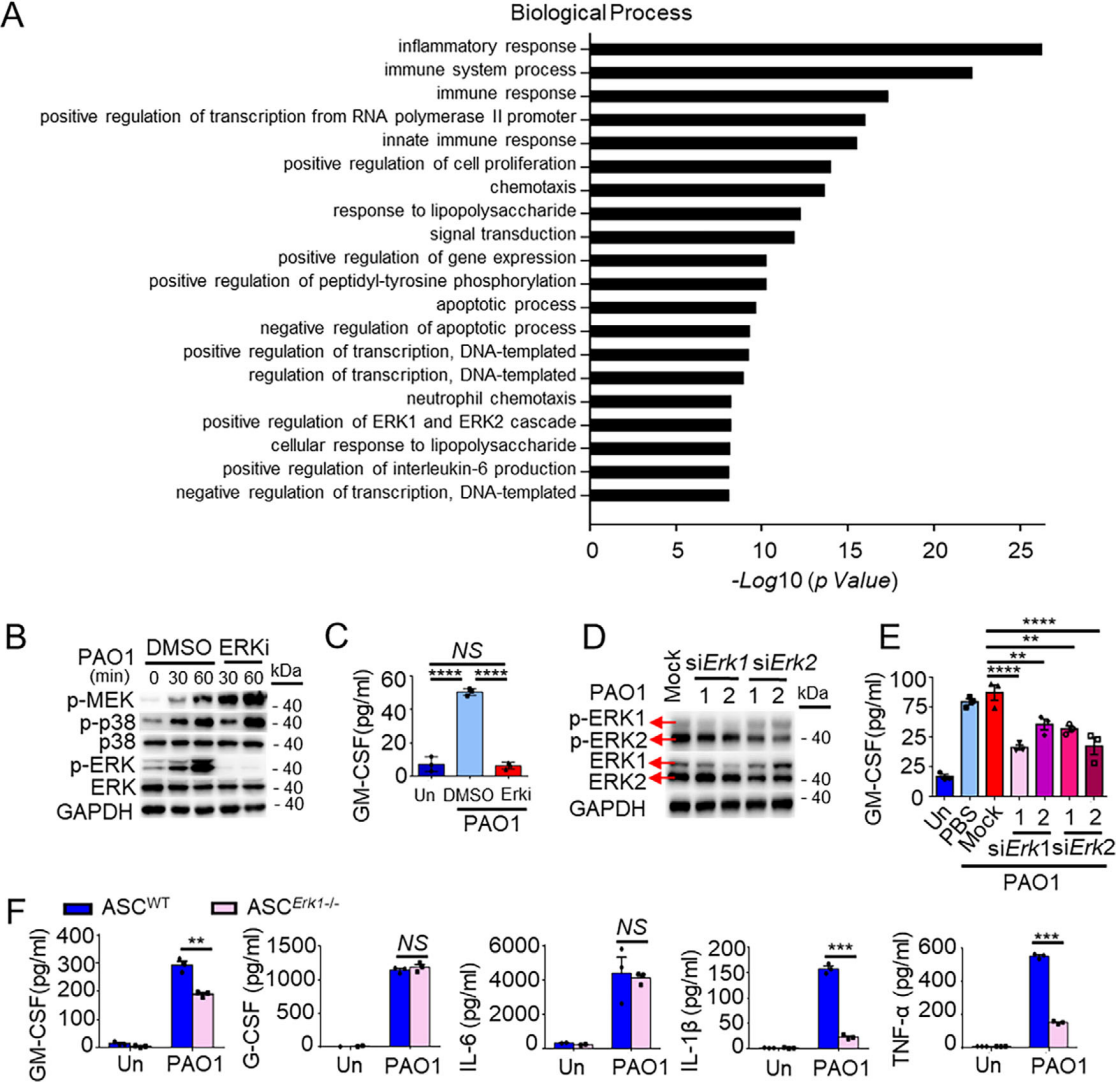
Extracellular regulated protein kinases 1/2 (ERK1/2) are critical regulators of granulocyte‐macrophage colony‐stimulating factor (GM‐CSF) secretion in adipose tissue‐derived mesenchymal stem cells (ASCs). (A) The biological process of gene ontology analysis was generated from microarray analysis data in ASCs after stimulation with PBS (Un) or PAO1 (MOI = 1) for 4 h (*n* = 3/group). (B) Western blot analysis of phosphorylated (p‐) and total MEK, p38, ERK1/2 and GAPDH (loading control, left margin), in ASCs, pretreated with the vehicle DMSO or with ERK1/2 inhibitors (ERKi; U0126‐Etoh, 10 μM, above lanes) and stimulated with PAO1 for 0–60 min (above blots; MOI = 1). (C) ELISA results for GM‐CSF in supernatants of ASCs treated with the vehicle DMSO or ERKi and stimulated with PAO1 (MOI = 1) for 8 h. (*n* = 3/group). (D, E) ASCs were transfected with small interfering RNA (siRNA) for ERK1, ERK2, or non‐target dsRNA (Mock; above lanes). Relative protein expression of p‐ and total ERK1/2 and GAPDH (loading control, left margin) in ASCs stimulated with PAO1 for 60 min was determined by Western blot (D) and GM‐CSF in supernatants of ASCs stimulated with PAO1 (MOI = 1) for 8 h was determined by ELISA (*n* = 3/group) (E). (F) ELISA results for GM‐CSF, G‐CSF, IL‐6, IL‐1β, and TNF‐α in supernatants of WT‐ASCs and *Erk1* ^−/−^ ‐ASCs stimulated with PAO1 at MOI = 1 for 8 h (*n* = 3/group). Statistical difference calculated by one‐way ANOVA with *Tukey's* multiple comparisons test (C, E), two‐tailed unpaired *t*‐test (F). Results are presented as mean ± SEM and are representative of three independent experiments. ^**^ *p *< 0.01, ^***^ *p *< 0.005, ^****^ *p *< 0.001; *NS*, not significant

Ontology assay from Microarray data showed that *P. aeruginosa* stimulation induced a significant change in the group of positive regulation of ERK1 and ERK2 cascade (Figure 6A), suggesting that ERK1/2 might be critical for ASCs against *P. aeruginosa* infection. Notably, inhibition of ERK1/2 fully eliminated *P. aeruginosa*‐mediated GM‐CSF secretion of ASCs (Figure 6B,C). In addition, by using RNA interference, knockdown of ERK1 or ERK2 could also significantly decrease *P. aeruginosa*‐induced GM‐CSF secretion (Figure 6D,E), whereas knockdown of signal transducer and activator of transcription 3 (Stat3), Spleen tyrosine kinase (Syk), or Raf1 showed no inhibitory effect on GM‐CSF secretion (Figure S5A–D). Consistently, *Erk1* ^−/−^‐ASCs expressed a lower level of GM‐CSF by 35.13%. Moreover, *Erk1* deficiency significantly reduced the secretion of IL‐1β by 85.33% and TNF‐α by 72.19% compared with WT‐ASCs responding to *P. aeruginosa* (Figure 6F). These data suggested that ERK1/2 were regulators for GM‐CSF production in ASCs.

### 3.7 CD69 mediates GM‐CSF secretion via ERK1 but not ERK2

**FIGURE 7:**
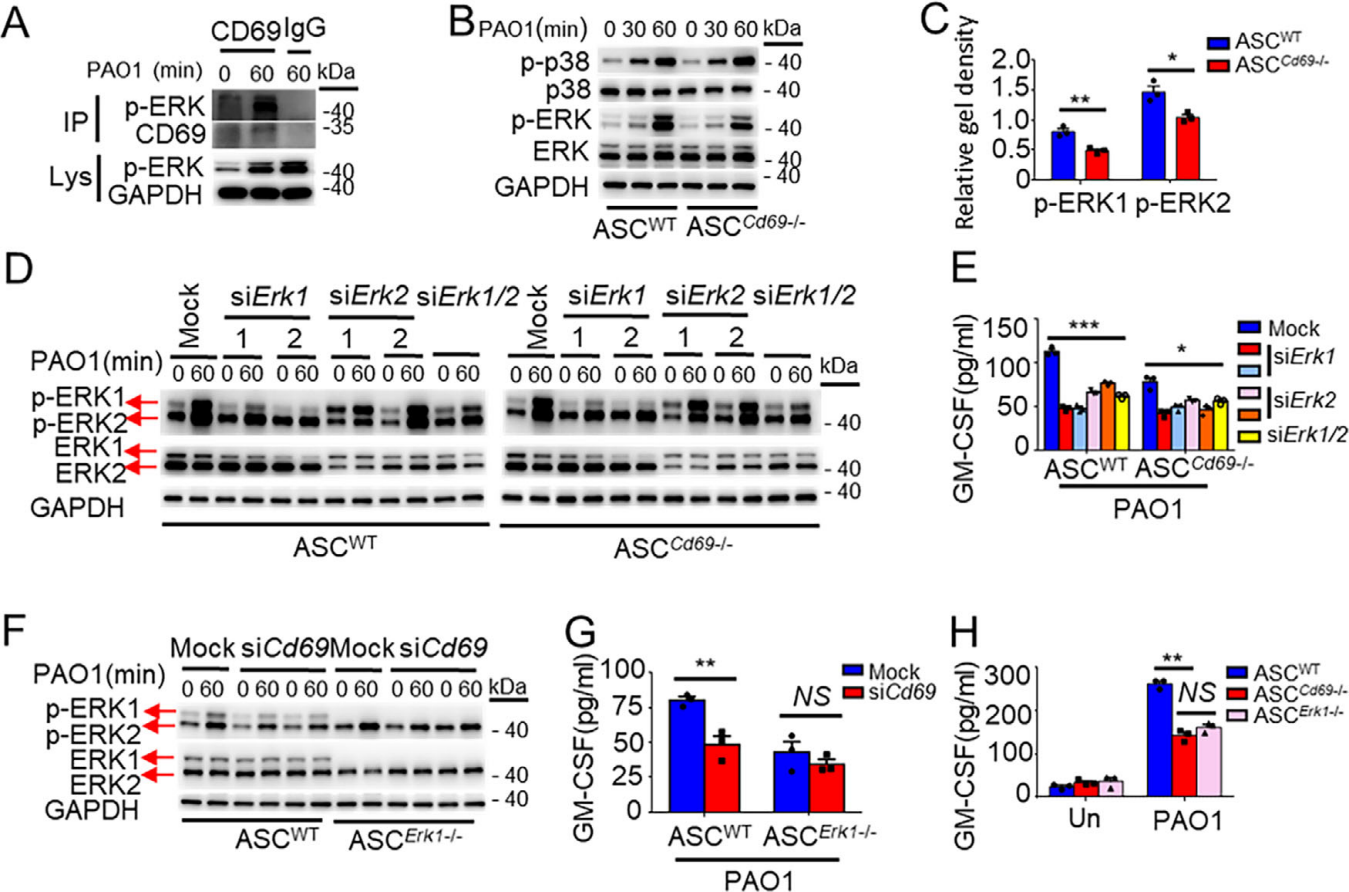
CD69 mediates granulocyte‐macrophage colony‐stimulating factor (GM‐CSF) secretion via extracellular regulated protein kinases 1 (ERK1) but not ERK2. (A) Western blot analysis of the interaction of CD69 with p‐ERK1/2 (left margin) in WT‐ASCs stimulated with PAO1 for 0 or 60 min (above blots) (MOI = 1), assessing proteins immunoprecipitated (IP; left margin) from cell lysates with anti‐CD69 or IgG (IP Ab; above lanes) or in lysates without immunoprecipitation (Lys; left margin). (B, C) Western blot analysis of p‐ and total p38, ERK1/2, and GAPDH (loading control, left margin), in WT‐ASCs and *Cd69* ^−/−^‐ASCs (below lanes) stimulated with PAO1 for 0–60 min (above blots; MOI = 1). Quantification of p‐ERK1 and p‐ERK2 was performed on three individual samples. (D, E) WT‐ASCs and *Cd69* ^−/−^‐ASCs (below lanes) were transfected with siRNA for ERK1, ERK2, ERK1 plus ERK2, or non‐target dsRNA (Mock; above lanes). Relative protein expression of p‐ and total ERK1/2 and GAPDH (loading control, left margin) in adipose tissue‐derived mesenchymal stem cells (ASCs) stimulated with PAO1 for 0 or 60 min (above blots) was determined by western blot (D) and GM‐CSF in supernatants of ASCs stimulated with PAO1 (MOI = 1) for 8 h was determined by ELISA (*n* = 3/group) (E). (F, G) WT‐ASCs and *Erk1* ^−/−^‐ASCs (below lanes) were transfected with siRNA for CD69 or non‐target dsRNA (Mock; above lanes). Relative protein expression of p‐ and total ERK1/2 and GAPDH (loading control) (left margin) in ASCs stimulated with PAO1 for 0 or 60 min (above blots) was determined by western blot (F) and GM‐CSF in supernatants of ASCs stimulated with PAO1 (MOI = 1) for 8 h was determined by ELISA (*n* = 3/group) (G). (H) ELISA results for GM‐CSF in supernatants of WT‐ASCs, *Cd69* ^−/−^‐ASCs and *Erk1* ^−/−^ ‐ASCs stimulated with PAO1 at MOI = 1 for 8 h (*n* = 3/group). Statistical difference calculated by one‐way ANOVA with *Tukey's* multiple comparisons test (E, H), two‐tailed unpaired *t*‐test (C, G). Results are presented as mean ± SEM and are representative of three independent experiments. ^*^ *p *< 0.05, ^**^ *p *< 0.01, ^***^ *p *< 0.005; *NS*, not significant

We subsequently explored the association between CD69 and ERK1/2 via co‐immunoprecipitation and found that CD69 could interact with p‐ERK1/2 in ASCs after *P. aeruginosa* stimulation (Figure 7A). Furthermore, CD69 deficiency could inhibit *P. aeruginosa*‐induced ERK1/2 phosphorylation instead of p38 phosphorylation (Figure 7B). Notably, depletion of CD69 significantly reduced the levels of ERK1 phosphorylation by 38.97%, but by 28.78% of the levels of ERK2 phosphorylation (Figure 7C). All the findings suggested that CD69 might interact with p‐ERK1 directly to regulate the function of ASCs in response to *P. aeruginosa* infection.

To investigate whether CD69 facilitates ERK1 activation to increase the GM‐CSF secretion in ASCs, we knocked down either ERK1 or ERK2 by siRNA in WT‐ and *Cd69^−/−^*‐ASCs, and measured the production of GM‐CSF in ASCs. The results showed that knockdown of ERK1 could significantly decrease p‐ERK1 and p‐ERK2 while knockdown of ERK2 by siRNA could only significantly decrease p‐ERK2 (Figure 7D). Also, we found that knockdown of p‐ERK1/2 or p‐ERK2 could decrease the production of GM‐CSF in *Cd69^−/−^*‐ASCs (Figure 7E). These results suggested that ERK2 was dispensable for CD69 mediated GM‐CSF secretion in ASCs. For the next step, when knocking down CD69 by siRNA in WT‐ and *Erk1^−/−^*‐ASCs, no effect could be found on GM‐CSF secretion in *Erk1^−/−^*‐ASCs response to *P. aeruginosa* (Figure 7F,G), indicating that ERK1 was the key downstream signaling protein for CD69 to regulate GM‐CSF secretion. Moreover, deficiency of *Cd69* and *Erk1* represented similar effects on GM‐CSF secretion (Figure 7H). Thus, we determined that CD69 facilitated GM‐CSF secretion through the activation of ERK1 in ASCs stimulated with *P. aeruginosa*.

### 3.8 ERK1 deficiency completely abolishes the protective role of ASCs against *P. aeruginosa* pneumonia

**FIGURE 8:**
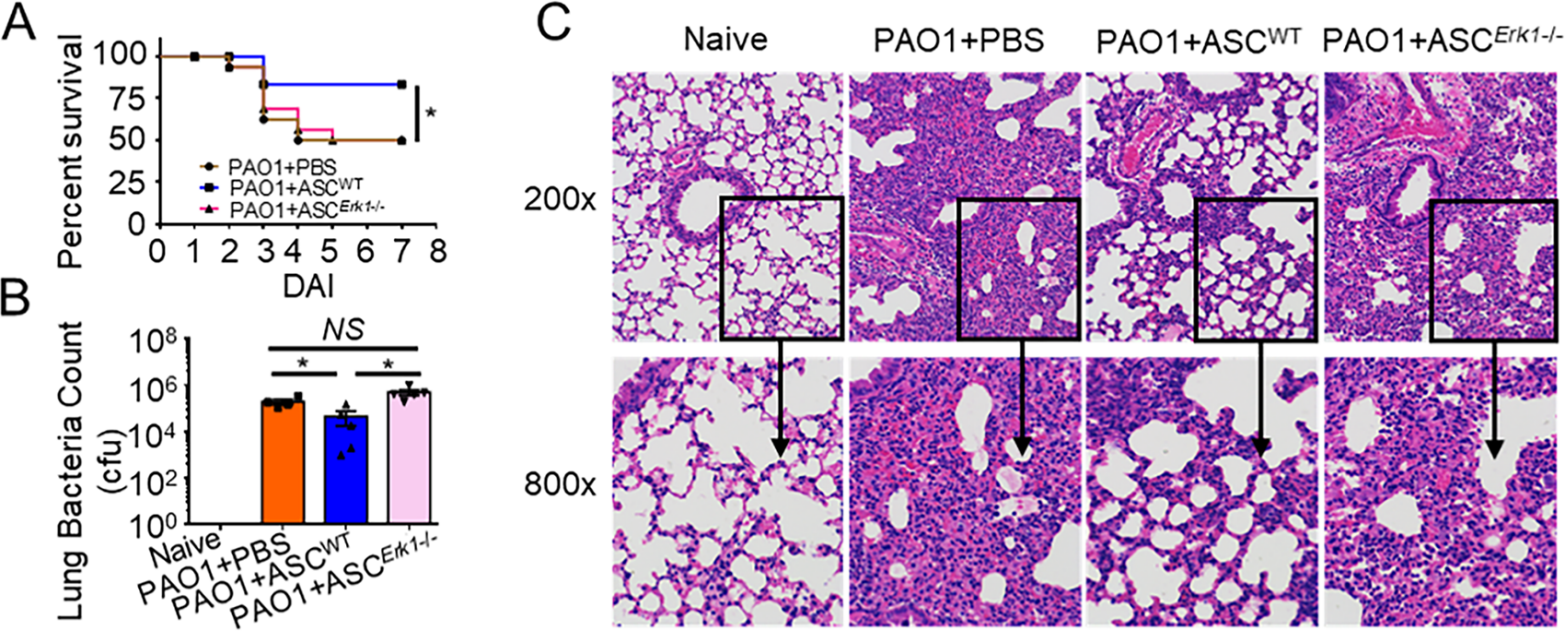
Extracellular regulated protein kinases 1 (ERK1) deficiency completely abolishes the protective role of ASCs against *Pseudomonas aeruginosa* (*P. aeruginosa*) pneumonia. (A–C) Mice were infected with 3 × 10^6^ CFU PAO1 by i.t., then given therapeutic delivery of PBS, 1 × 10^6^ WT‐ASCs or *Erk1* ^−/−^‐ASCs by i.t. 4 h later, respectively. (A) Survival of mice receiving PBS (*n* = 16), WT‐ASCs (*n* = 15), or *Cd69* ^−/−^‐ASCs (*n* = 16) for 7 days. (B) CFU assay of lung tissue homogenate at 24 hpi (*n* = 5/group); (C) Representative pathological sections of lungs analyzed with hematoxylin and eosin (H&E) staining at 24 hpi. Statistical difference calculated by one‐way ANOVA with *Tukey's* multiple comparisons test (B), or *log‐rank* (*Mantel‐Cox*) test (A). Results are presented as mean ± SEM and are representative of three independent experiments. ^*^ *p *< 0.05; *NS*, not significant

To determine the effects of ERK1 in ASCs against *P. aeruginosa* pneumonia, we used PBS, WT‐ASCs, or *Erk1* ^−/−^‐ASCs to treat *P. aeruginosa* pneumonia mice. *Erk1* ^−/−^‐ASCs treatment group presented higher mortality (Figure 8A), bacterial burden (Figure 8B), and severity of lung injury (Figure 8C) compared with the WT‐ASCs treatment group, while there were no significant differences between the PBS control group and *Erk1* ^−/−^‐ASCs treated group. Together, the effects of *Erk1* ^−/−^‐ASCs were similar to the administration of *Cd69^−/−^*‐ASCs, which indicated that the CD69‐ERK1 pathway played a key role in ASCs‐based therapy against *P. aeruginosa* pneumonia.

## 4 DISCUSSION

**FIGURE 9:**
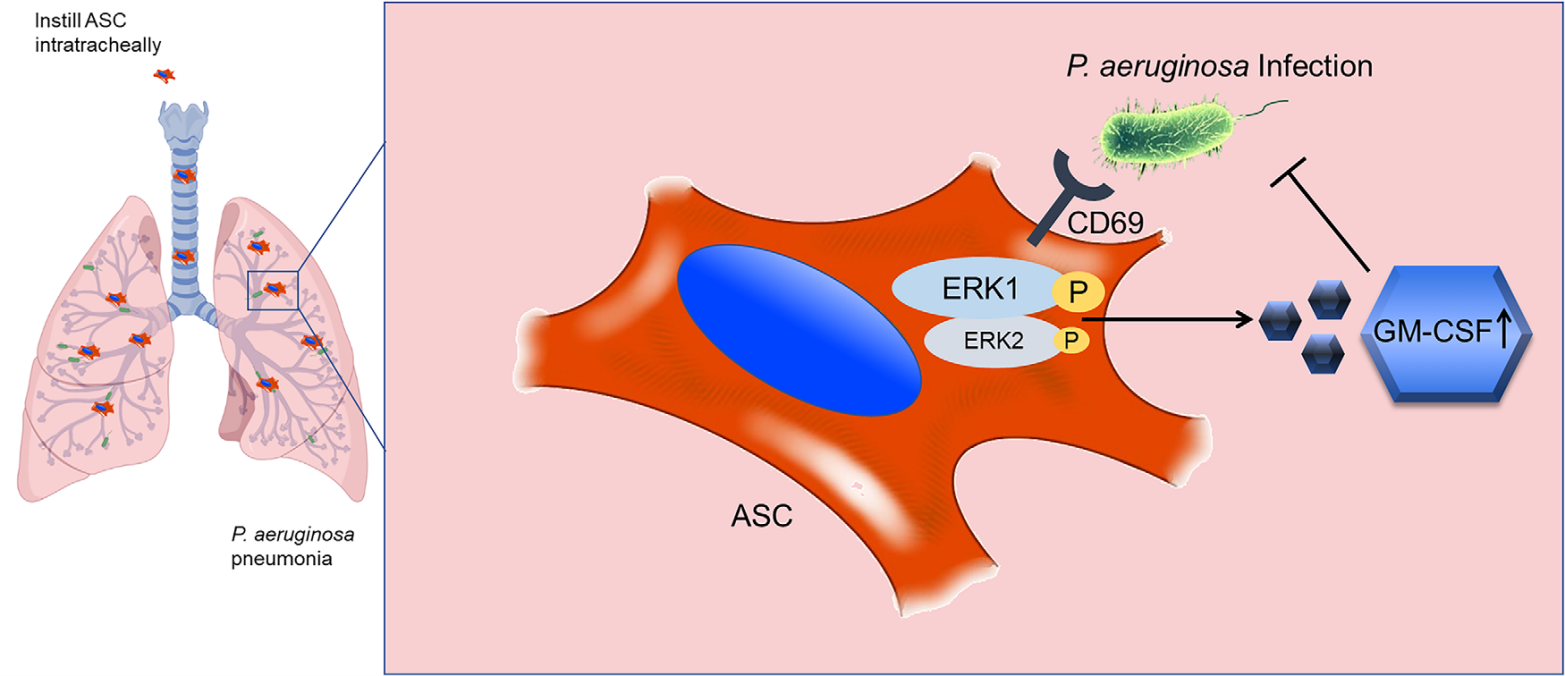
Graphical representation of CD69‐regulated protective role of ASCs against *Pseudomonas aeruginosa* (*P. aeruginosa*) pneumonia. The expression of *Cd69* in adipose tissue‐derived mesenchymal stem cells (ASCs) was significantly upregulated during *P. aeruginosa* infection, thereby facilitating the recognition of *P. aeruginosa* by CD69. After this recognition, CD69 activated the extracellular regulated protein kinases 1 (ERK1) pathway and promoted the granulocyte‐macrophage colony‐stimulating factor (GM‐CSF) secretion of ASCs, which finally promoted the alleviation of *P. aeruginosa* pneumonia

In this study, we found that CD69 was critical for ASCs‐mediated benefits in *P. aeruginosa* pneumonia. Mechanistically, CD69 recognized *P. aeruginosa* to promote ERK1 activation in ASCs upon *P. aeruginosa* challenge, followed by GM‐CSF and other inflammatory cytokines secretion. The deficiency of CD69 or ERK1 dramatically decreased GM‐CSF production and eliminated the therapeutic effect of ASCs in *P. aeruginosa* pneumonia (Figure 9). We firstly reported that CD69, a C‐type lectin receptor expressed on ASCs, could recognize *P. aeruginosa* to promote ERK1 activation, which modulated ASCs functions. These findings provided a novel insight for ASCs‐mediated protection from *P. aeruginosa* pneumonia.

Previous researches on CD69 only focused on its function among immune cells, such as T cells and NK cells.^22^, ^23^, ^24^ The whole profile of CD69 needs to be clarified. Our study firstly reported that CD69 functioned as an important receptor in ASCs in response to *P. aeruginosa*. Existing evidence showed that CD69 was a complex immunoregulatory receptor, which depended on the cellular context and its ligand(s).^18^, ^21^ In our study, we found that ASCs could express CD69 to recognize *P. aeruginosa* and regulate some inflammatory cytokine secretion. A previous study showed that CD69 is a C‐type lectin receptor that could bind with soluble recombinant Gal‐1.^18,21^ In the present, we showed that the binding of CD69 with *P. aeruginosa* could be competitively inhibited by Gal‐1. Furthermore, F98 and I99 would be the most important binding site confirmed by constructing hFc‐hCD69 recombinant protein. Moreover, deficiency of CD69 dramatically decreased the production of GM‐CSF, IL‐1β, and TNF‐α on ASCs, and failed to protect mice against *P. aeruginosa* pneumonia, also indicating the critical role of CD69 in the functional regulation of ASCs, especially in anti‐*P. aeruginosa* infection. Notably, our study also showed that administration of GM‐CSF could increase bacteria clearance effects of *Cd69^−/−^*‐ASCs, which demonstrated that CD69 mediated the protective effects of ASCs at least partly through regulating GM‐CSF secretion.

Mitogen‐activated protein kinase family was pivotal to regulate growth factors expression in response to numerous environmental stimuli,^25^, ^26^ and ERK1/2 served as central signaling molecules for GM‐CSF production in many cell types, such as macrophages,^27^ thymic epithelial cells,^28^ and bronchial epithelial cells.^29^ Our study showed that activation of ERK1/2 is necessary for *P. aeruginosa*‐induced GM‐CSF in ASCs. In addition, our study found that CD69 deficiency could inhibit *P. aeruginosa*‐induced ERK1/2 phosphorylation, which was consistent with other findings demonstrating that CD69 could activate ERK1/2 to mediate cell degranulation in NK cells.^30^ However, the inhibition of CD69 deficiency on ERK1 phosphorylation was stronger than ERK2 phosphorylation, and knockdown of CD69 in *Erk1^−/−^*‐ASCs could not affect GM‐CSF secretion. Thus, we concluded that ERK1 was the key downstream signaling protein for CD69 to upregulated the production of GM‐CSF on ASCs in response to *P. aeruginosa* infection. Moreover, deficiency of ERK1 dramatically decreased the production of GM‐CSF, IL‐1β, and TNF‐α on ASCs, and failed to protect mice against *P. aeruginosa* pneumonia, which was in line with the effect of CD69 deficiency. All these data above indicated that ERK1 was a critical regulator of CD69 signaling in ASCs.

GM‐CSF could be produced by various cell series including fibroblasts, endothelial cells, macrophages, mast cells, and T cells.^31^, ^32^, ^33^ However, no existing evidence revealed the relationship between ASCs and GM‐CSF. Our study showed that in response to the *P. aeruginosa* challenge, ASCs could secret a high level of GM‐CSF both in vitro and in vivo, in line with previous studies suggesting that human bone‐marrow‐derived mesenchymal stem/stromal cells could upregulate transcription of GM‐CSF under the stimulation of Concanavalin‐A.^20^ To date, GM‐CSF was demonstrated protective potentiality against infection including *S. pneumoniae* infection, *P. aeruginosa* infection, Lethal Influenza infection, and sepsis.^34^, ^35^, ^36^, ^37^, ^38^ Mesenchymal stem/stromal cells were established to release paracrine factors, microvesicles, and transfer mitochondria.^39^, ^40^, ^41^ Consistent with the findings above, our study indicated that the therapeutic effect of ASCs on *P. aeruginosa* pneumonia was at least partially through the secretion of GM‐CSF.

Although we found that CD69 could bind to *P. aeruginosa*, we did not figure out the ligand on *P. aeruginosa*. Future studies will need to identify the specific ligands on *P. aeruginosa*. Given the pivotal role of CD69 in ASCs against *P. aeruginosa* pneumonia, further investigation is required to modify the function of ASCs with CD69 as the target for improving the therapeutic efficacy. CD69 can be expressed on activated lymphocytes, infiltrated leukocytes, and endothelial cells,^6^, ^7^, ^8^, ^9^ but the role of CD69 in host defense against *P. aeruginosa* infection was rarely reported. Our study showed the importance of CD69 in recognizing *P. aeruginosa* and regulating the therapeutic role of ASCs. In light of these observations, we reason that CD69 may play a role in host defense against *P. aeruginosa* infection, and this hypothesis deserves further investigation.

In summary, our study firstly demonstrated that ASCs protected mice against *P. aeruginosa* pneumonia via expressing CD69 for recognizing *P. aeruginosa* to facilitate ERK1 activation. Our findings revealed a novel insight into the role of CD69 as well as provided a potential candidate for the effective treatment of *P. aeruginosa* infection.

## AUTHOR CONTRIBUTIONS

Yanshan Jiang designed and performed experiments, analyzed data, and wrote the paper. Fan Li designed and performed some experiments. Yanan Li performed some experiments and analyzed data. Jielin Duan designed and performed some experiments. Caixia Di designed some experiments and wrote the paper. Yinggang Zhu and Jingya Zhao wrote the paper. Xinming Jia and Jieming Qu initiated the study, organized, designed, and wrote the paper.

## CONFLICT OF INTEREST

The authors declare that they have no conflict of interest.

## Supporting information

Supporting information

Supporting information

Supporting information

Supporting information

Supporting information

Supporting information

Supporting information

## Data Availability

All the data supporting the findings of this study are available within the article and its supplementary information files and from the corresponding author upon reasonable request.
