# Supplementary material for: CD69 mediates the protective role of adipose tissue‐derived mesenchymal stem cells against Pseudomonas aeruginosa pulmonary infection
Source: Clin Transl Med. 2021 Nov 4;11(11):e563. doi: 10.1002/ctm2.563 (PMC8567058; doi:10.1002/ctm2.563)
Supplement: Supplementary file 1 — Supporting information [file CTM2-11-e563-s005.docx]

Supplementary Materials for

CD69 mediates the protective role of adipose tissue-derived mesenchymal stem cells against *Pseudomonas aeruginosa* pulmonary infection

Yan-Shan Jiang, Fan Li, Ya-Nan Li, Jie-Lin Duan, Cai-Xia Di, Ying-Gang Zhu, Jing-Ya Zhao, Xin-Ming Jia, Jie-Ming Qu

Correspondence to: jmqu0906@163.com (J.M.Q.) or jiaxm@tongji.edu.cn (X.M.J.)

**This file includes:**

Figure S1, related to Figure 1

Figure S2, related to Figure 2

Figure S3, related to Figure 3

Figure S4, related to Figure 4

Figure S5, related to Figure 6

Figure S6, Identification of *GM-CSF*^-/-^ mice, *Erk1*^-/-^ mice and *Cd69*^-/-^ mice.

Table S1, Oligonucleotides used in this study.

Table S2. Key resources table.

Supplementary methods

**Figure S1, related to Figure 1. GM-CSF secreted by ASCs exhibits the protective role against *P. aeruginosa* pneumonia.**

(A) Molecular Function of Gene ontology (GO) analysis generated from Microarray analysis data in ASCs after stimulation with PBS (Un) or PAO1 (MOI=1) for 4 hours (n=3/group).

(B) Protocol of acute *P. aeruginosa* pneumonia and ASCs therapy in mice. WT male mice (C57BL/6J) were infected with 3 × 10^6^ CFU PAO1 by i.t., then given therapeutic delivery of 1 × 10^6^ ASCs by i.t. 4 hours later and sacrificed at 24 hpi.

(C) Protocol of FACS for GFP positive ASCs (GFP-ASCs) distributed within the lung. WT male mice (C57BL/6J) infected with 3 × 10^6^ CFU PAO1 or PBS by intratracheal (i.t.) administration, were treated with GFP-ASCs by i.t. 4 hours later, and then were sacrificed 8 hours post infection (hpi). The lung was prepared to enrich GFP-ASCs by FACS.

**Figure S2, related to Figure 2.** **GM-CSF secretion is independent of TLR2, TLR4, NLRP3 or NLRC4.**

(A, B) Total cell numbers of ASCs which had been stimulated with indicated bacteria (MOI=0.1, 1, and 5) for 8 hours.

**Figure S3, related to Figure 3. CD69 mediates GM-CSF and some inflammatory cytokines secretion of ASCs in response to *P. aeruginosa* challenge.**

(A) ELISA results for GM-CSF in supernatants of WT-ASCs, *Clec7a*^-/-^-ASCs, or *Clec4e*^-/-^-ASCs stimulated with PAO1 at MOI=1 for 8 hours (n=3/group).

(B-E) ASCs were transfected with siRNA or non- target dsRNA (Mock). After 72 hours, relative protein expression of OLR1 (B) and MDA5 (D) and GAPDH (loading control) in ASCs was determined by Western blot, meanwhile GM-CSF in supernatants of ASCs transfected with siRNA for Olr1 (C), MDA5 (E) stimulated with PAO1 (MOI=1) for 8 hours was determined by ELISA (n=3/group).

Statistical difference calculated by one-way *ANOVA* with *Tukey's* multiple comparisons test (A, C and E). Results are presented as mean ± SEM and are representative of three independent experiments. ^*^, *p*<0.05; ^****^, *p*<0.001; *NS*, not significant.

**Figure S4, related to Figure 4. CD69 can specifically recognize *P. aeruginosa*.**

(A) Western blot analysis of soluble protein truncations of hCD69-hFc (above blots) purified from the supernatants of 293T cells transfected with plasmid vectors of hCD69-pFuse-hIgG1-Fc and its truncations for 3 days.

(B) The schematic pattern for all truncations of hCD69.

(C) Western blot analysis of the different point-mutated fusion proteins of hCD69-hFc purified from the supernatants of 293T cells transfected with corresponding plasmid vectors for 3 days. Met184, Leu190, Trp192, Ile193 were substituted with Alanine; Met184, Lys196, Lys199, Lys188 were substituted with Glutamic acid. All these mutations were located at 182-199 residues of hCD69.

(D) Western blot analysis of the different point-mutated fusion proteins of hCD69-hFc purified (above blots) from the supernatants of 293T cells transfected with corresponding plasmid vectors for 3 days. Phe98, Ile99, Ser100 were substituted with Alanine; Arg94 was substituted with Glutamic acid; All these mutations were located at 82-101 residues of hCD69.

(E) Flow cytometry was used to analyze the binding of the different point-mutated fusion proteins of hCD69-hFc to PAO1, whose mutations were located at 182-199 residues of hCD69. PAO1 was incubated with purified mutants for 1 hour, and stained with FITC-conjugated anti-Human IgG, Fcγ fragment specific antibody for 30 minutes.

Results are representative of three independent experiments.

**Figure S5, related to Figure 6. ERK1/2 are critical regulators of GM-CSF secretion in ASCs.**

(A, B) ASCs were transfected with siRNA for Stat3 or non-target dsRNA (Mock) (above lanes). Relative protein expression of phosphorylated (p-) and total Stat3 and Actin (loading control) (left margin) in ASCs stimulated with PAO1 for 0 or 60 minutes (above blots) was determined by Western blot (A), GM-CSF in supernatants of ASCs stimulated with PAO1 (MOI=1) for 8 hours was determined by ELISA (n=3/group) (B).

(C, D) ASCs were transfected with siRNA for Syk, Raf1 or non- target dsRNA (Mock) (above blots). Relative protein expression of Syk, Raf1 and GAPDH (loading control) (left margin) in ASCs was determined by Western blot (C) and GM-CSF in supernatants of ASCs stimulated with PAO1 (MOI=1) for 8 hours was determined by ELISA (n=3/group) (D).

Statistical difference calculated by one-way *ANOVA* with *Tukey's* multiple comparisons test (B and D). Results are presented as mean ± SEM and are representative of three independent experiments. ^***^, *p*<0.005; *NS*, not significant.

**Figure S6.** **Identification of *GM-CSF*^-/-^ mice, *Erk1*^-/-^ mice and *Cd69*^-/-^ mice.**

(A) Identification of the genotype of WT mice and *GM-CSF* deficient (*GM-CSF*^-/-^) mice by PCR analysis. Mutant=~300bp; Wild type=317bp.

(B) ELISA results for GM-CSF in supernatants of WT-ASCs and *GM-CSF*^-/-^-ASCs stimulated with PAO1 at MOI=1 for 8 hours (n=3/group). UD, undetected.

(C) PCR analysis of the genotype of WT mice, *Erk1^+/-^* mice and *Erk1^-/-^* mice. Mutant=~398bp; Wild type=4125bp and 243bp; Heterozygote=~398bp and 243bp.

(D) Western blot analysis of p- and total ERK1/2 and IκBα and GAPDH (loading control) (left margin), in WT-ASCs and *Erk1*^-/-^-ASCs (below lanes) stimulated with PAO1 for 0–60 minutes (above blots) (MOI=1).

(E) PCR analysis of the genotype of WT mice, *Cd69^+/-^* mice and *Cd69^-/-^* mice. Mutant=332bp; Wild type=422bp; Heterozygote=322bp and 422bp.

(F) Western blot analysis of CD69 and GAPDH (loading control) (left margin) in thymus, bone marrow, spleen, liver, kidney and lung (below lanes) from WT mice and *Cd69*^-/-^ mice (above blots).

(G) Quantitative real-time PCR analysis of the *CD69* in WT-ASCs and *Cd69*^-/-^-ASCs stimulated with PAO1 (MOI=1) for 0, 2 or 4 hours; results were normalized to those of the control gene *β-actin* (n=3/group).

Results are presented as mean ± SEM and are representative of three independent experiments.

**Table S1. Oligonucleotides used in this study.**

| **Primer sequences for amplification of the extracellular domain of hCD69** | | |
| --- | --- | --- |
| NO. | Gene | Sequence 5’～3’ |
| 1 | h-CD69-hFc | F AGTCTTGCACTTGTCACGAATTCGTCAGTGGGCCAATACAATTG |
|  |  | R GCATGTGTGAGT TTTGTCAGATCTTTTGTAAGGTTTGTTACATA |
| **Primer sequences for amplification of truncations** | | |
| NO. | Truncations | Sequence 5’～3’ |
| 1 | 82-199aa | F AGTCTTGCACTTGTCACGAATTCGGTTTCTTCATGCTCTGAGGA |
|  |  | R GCATGTGTGAGT TTTGTCAGATCTTTTGTAAGGTTTGTTACATA |
| 2 | 102-199aa | F AGTCTTGCACTTGTCACGAATTCGGTGAAGAGGAGCTGGACTTC |
|  |  | R GCATGTGTGAGT TTTGTCAGATCTTTTGTAAGGTTTGTTACATA |
| 3 | 122-199aa | F AGTCTTGCACTTGTCACGAATTCGGTCATTGATTCTGAAAAGGA |
|  |  | R GCATGTGTGAGT TTTGTCAGATCTTTTGTAAGGTTTGTTACATA |
| 4 | 144-199aa | F AGTCTTGCACTTGTCACGAATTCGTGGGTTGGACTGAAAAAGGA |
|  |  | R GCATGTGTGAGT TTTGTCAGATCTTTTGTAAGGTTTGTTACATA |
| 5 | 162-199aa | F AGTCTTGCACTTGTCACGAATTCGAACAACTGGTTCAACGTTAC |
|  |  | R GCATGTGTGAGT TTTGTCAGATCTTTTGTAAGGTTTGTTACATA |
| 6 | 182-199aa | F AGTCTTGCACTTGTCACGAATTCGAGCAGCATGGAATGTGAGAA |
|  |  | R GCATGTGTGAGT TTTGTCAGATCTTTTGTAAGGTTTGTTACATA |
| **Primer sequences for amplification of mutations** | | |
| NO. | Mutations | Sequence 5’～3’ |
| 1 | F98A | F CTACCAGAGGAAATGCTACGCTATTTCTACTGTGAAGAGGAG |
|  |  | R CTCCTCTTCACAGTAGAAATAGCGTAGCATTTCCTCTGGTAG |
| 2 | I99A | F CCAGAGGAAATGCTACTTTGCTTCTACTGTGAAGAGGAGCTG |
|  |  | R CAGCTCCTCTTCACAGTAGAAGCAAAGTAGCATTTCCTCTGG |
| 3 | S100A | F GAAATGCTACTTTATTGCTACTGTGAAGAG |
|  |  | R CAATAAAGTAGCATTTCCTCTGGTAGCC |
| 4 | R94E | F GACTGGGTTGGCTACCAGGAGAAATGCTACTTTATTTCTAC |
|  |  | R GTAGAAATAAAGTAGCATTTCTCCTGGTAGCCAACCCAGTC |
| 5 | M184A | F GAAAAACACAGAGGTCAGCAGCGCGGAATGTGAGAAGAATTTATAC |
|  |  | R GTATAAATTCTTCTCACATTCCGCGCTGCTGACCTCTGTGTTTTTC |
| 6 | L190A | F GCATGGAATGTGAGAAGAATGCATACTGGATATGTAACAAAC |
|  |  | R GTTTGTTACATATCCAGTATGCATTCTTCTCACATTCCATGC |
| 7 | W192A | F GAATGTGAGAAGAATTTATACGCGATATGTAACAAACCTTAC |
|  |  | R GTAAGGTTTGTTACATATCGCGTATAAATTCTTCTCACATTC |
| 8 | I193A | F GAATGTGAGAAGAATTTATACTGGGCATGTAACAAACCTTAC |
|  |  | R GTAAGGTTTGTTACATGCCCAGTATAAATTCTTCTCACATTC |
| 9 | W192A &I193A | F GAATGTGAGAAGAATTTATACGCGGCATGTAACAAACCTTAC |
|  |  | R GTAAGGTTTGTTACATGCCGCGTATAAATTCTTCTCACATTC |
| 10 | L190A &M184A | F GCGCGGAATGTGAGAAGAATGCATACTGGATATGTAACAAAC |
|  |  | R GTTTGTTACATATCCAGTATGCATTCTTCTCACATTCCGCGC |
| 11 | M184E | F GAAAAACACAGAGGTCAGCAGCGAGGAATGTGAGAAGAATTTATAC |
|  |  | R GTATAAATTCTTCTCACATTCCTCGCTGCTGACCTCTGTGTTTTTC |
| 12 | L190E | F GCATGGAATGTGAGAAGAATGAATACTGGATATGTAACAAAC |
|  |  | R GTTTGTTACATATCCAGTATTCATTCTTCTCACATTCCATGC |
| 13 | K196E | F GAATTTATACTGGATATGTAACGAACCTTACAAAAG |
|  |  | R CGTTACATATCCAGTATAAATTCTTCTCACATTC |
| 14 | K199E | F GATATGTAACAAACCTTACGAAAGATCTGAC |
|  |  | R CGTAAGGTTTGTTACATATCCAGTATAAATTC |
| 15 | K188E | F CAGCAGCATGGAATGTGAGGAGAATTTATACTGG |
|  |  | R CCTCACATTCCATGCTGCTGACCTCTGTG |
| **Primer sequences for siRNA** | | |
| NO. | Gene | Sequence 5’～3’ |
| 1 | *Cd69* | F CCAUGGCACCAGUAUACAUTT |
|  |  | R AUGUAUACUGGUGCCAUGGTT |
| 2 | *Olr1-1* | F GGUGCAGAUGAUCUGACAUTT |
|  |  | R AUGUCAGAUCAUCUGCACCTT |
| 3 | *Olr1-2* | F GGAUUGGAUUGCAUCGGAATT |
|  |  | R UUCCGAUGCAAUCCAAUCCTT |
| 4 | *Olr1-3* | F GCGUUUCUUUACAGCUAUATT |
|  |  | R UAUAGCUGUAAAGAAACGCTT |
| 5 | *MDA5* | F CCUACAAAUCAACGACACGAU |
|  |  | R AUCGUGUCGUUGAUUUGUAGG |
| 6 | *Erk1*-1 | F CUCAACCACAUUCUAGGUATT |
|  |  | R UACCUAGAAUGUGGUUGAGTT |
| 7 | *Erk1*-2 | F GAACAGUACUACGAUCCGATT |
|  |  | R UCGGAUCGUAGUACUGUUCTT |
| 8 | *Erk2*-1 | F GUGAUCUCAAGAUCUGUGATT |
|  |  | R UCACAGAUCUUGAGAUCACTT |
| 9 *Erk2*-2 | | F CAACCAUUGAGCAAAUGAATT |
|  |  | R UUCAUUUGCUCAAUGGUUGTT |
| 10 *Stat3-1* | | F CCCGCCAACAAAUUAAGAATT |
|  |  | R UUCUUAAUUUGUUGGCGGGTT |
| 11 *Stat3-2* | | F GGGUCUCGGAAAUUUAACATT |
|  |  | R UGUUAAAUUUCCGAGACCCTT |
| 12 *Stat3-3* | | F GAGGAGGCAUUUGGAAAGUTT |
|  |  | R ACUUUCCAAAUGCCUCCUCTT |
| 13 *Raf-1* | | F GCAGCAGUCUCUACAAACATT |
|  |  | R UGUUUGUAGAGACUGCUGCTT |
| 14 *Raf-2* | | F GCGGAAUGGAAUGAGCUUATT |
|  |  | R UAAGCUCAUUCCAUUCCGCTT |
| 15  *Raf-3* | | F CGAGUUAUUACUGGGAAAUTT |
|  |  | R AUUUCCCAGUAAUAACUCGTT |
| 16 *Syk-1* | | F CCUGCUGCACGAAGGGAAATT |
|  |  | R UUUCCCUUCGUGCAGCAGGTT |
| 17 *Syk-2* | | F GGAUCAAAUCCUACUCCUUTT |
|  |  | R AAGGAGUAGGAUUUGAUCCTT |
| 18 *Syk-3* | | F GGAGCGAAGUGACCGCCAUTT |
|  |  | R AUGGCGGUCACUUCGCUCCTT |
| **Primer sequences for Quantitative real-time PCR** | | |
| NO. | Gene | Sequence 5’～3’ |
| 1 | *β-actin* | F GGCTGTATTCCCCTCCATCG |
|  |  | R CCAGTTGGT AACAATGCCATGT |
| 2 | *GM-CSF* | F GGCCTTGGAAGCATGTAGAGG |
|  |  | R GGAGAACTCGTTAGAGACGACTT |
| 3 | *G-CSF* | F ATGGCTCAACTTTCTGCCCAG |
|  |  | R CTGACAGTGACCAGGGGAAC |
| 4 | *IL6* | F TAGTCCTTCCTACCCCAATTTCC |
|  |  | R TTGGTCCTTAGCCACTCCTTC |
| 5 | *Cxcl1* | F CTGGGATTCACCTCAAGAACATC |
|  |  | R CAGGGTCAAGGCAAGCCTC |
| 6 | *Lif* | F ATTGTGCCCTTACTGCTGCTG |
|  |  | R GCCAGTTGATTCTTGATCTGGT |
| 7 | *Areg* | F GGTCTTAGGCTCAGGCCATTA |
|  |  | R CGCTTATGGTGGAAACCTCTC |
| 8 | *Ereg* | F CTGCCTCTTGGGTCTTGACG |
|  |  | R GCGGTACAGTTATCCTCGGATTC |
| 9 | *Osm* | F ATGCAGACACGGCTTCTAAGA |
|  |  | R TTGGAGCAGCCACGATTGG |
| 10 | *Pdgfb* | F CATCCGCTCCTTTGATGATCTT |
|  |  | R GTGCTCGGGTCATGTTCAAGT |
| 11 | *Ngf* | F CCAGTGAAATTAGGCTCCCTG |
|  |  | R CCTTGGCAAAACCTTTATTGGG |
| 12 | *Sele* | F ATGCCTCGCGCTTTCTCTC |
|  |  | R GTAGTCCCGCTGACAGTATGC |
| 13 | *Nod2* | F CAGGTCTCCGAGAGGGTACTG |
|  |  | R GCTACGGATGAGCCAAATGAAG |
| 14 | *Cd69* | F AGGCTTGTACGAGAAGTTGGA |
|  |  | R AGTTCACCAGAATATCGCTTCAG |
| 15 | *Clec4e* | F AGTGCTCTCCTGGACGATAG |
|  |  | R CCTGATGCCTCACTGTAGCAG |
| 16 | *Olr1* | F CAAGATGAAGCCTGCGAATGA |
|  |  | R ACCTGGCGTAATTGTGTCCAC |
| 17 | *Clec7a* | F GACTTCAGCACTCAAGACATCC |
|  |  | R TTGTGTCGCCAAAATGCTAGG |
| 18 | *Selp* | F CATCTGGTTCAGTGCTTTGATCT |
|  |  | R ACCCGTGAGTTATTCCATGAGT |
| 19 | *MDA5* | F AGATCAACACCTGTGGTAACACC |
|  |  | R CTCTAGGGCCTCCACGAACA |

**Table S2. Key resources table.**

| **Antibodys** | **Source** | | **Identifier** |
| --- | --- | --- | --- |
| FITC-Anti-Human IgG, Fcγ fragment specific | Jackson ImmunoResearch | | 109-095-008 |
| Alexa Fluor® 647-conjugated-Anti-Human IgG, Fcγ fragment specific | Jackson ImmunoResearch | | 109-605-008 |
| CD69 | Santa Cruz Biotechnology | | sc-373799 |
| phospho-ERK | Cell Signaling Technology | | 9101 |
| ERK | Cell Signaling Technology | | 4695 |
| phospho-p38 | Cell Signaling Technology | | 4511 |
| p38 | Cell Signaling Technology | | 8690 |
| phospho-IκBα | Cell Signaling Technology | | 9246 |
| IκBα | Cell Signaling Technology | | 4814 |
| MDA5 | Cell Signaling Technology | | 5321 |
| Olr1 | R&D | | AF1564 |
| phospho-Stat3(Ser727) | Cell Signaling Technology | | 34911 |
| phospho-Stat3(Tyr705) | Cell Signaling Technology | | 9145 |
| Stat3 | Cell Signaling Technology | | 12640 |
| Syk | Cell Signaling Technology | | 13198 |
| Raf1 | Cell Signaling Technology | | 53745 |
| CD69 (H1.2F3) | abcam | | ab25190 |
| Armenian Hamster IgG monoclonal (HTK888) | abcam | | ab18479 |
| **inhibitors** | **Source** | | **Identifier** |
| U0126-Etoh | Selleck | | S1102 |
| **Mice** | **Source** | **Genetic Background** | |
| wild-type mice | Charles River, **China** | C57BL/6J | |
| *GM-CSF*^−/−^ mice | The Jackson Laboratory | C57BL/6J | |
| *Tlr2*^−/−^mice | He’s lab ^1^ | C57BL/6J | |
| *Tlr4*^−/−^mice | He’s lab ^1^ | | C57BL/6J |
| *Clec4e*^−/−^mice | Jia’s lab | | C57BL/6J |
| *Clec7a*^−/−^ mice | Jia’s lab | | C57BL/6J |
| *Nlrp3*^−/−^ mice | Xu’s lab^2^ | | C57BL/6 |
| *Nlrc4*^−/−^ mice | Xu’s lab ^3^ | | C57BL/6 |
| *Cd69*^−/−^ mice | This study | | C57BL/6J |
| *Erk1*^−/−^ mice | This study | | C57BL/6J |
| **Strains** | **Source** | | **Identifier** |
| *PAO1* | ATCC | | BAA-47 |
| *GFP-PAO1* | Song’s lab ^4^ | | / |
| *MDR-PA* | ATCC | | BAA-2108 |
| *E.coli* | ATCC | | 25922 |
| *S.aureus* | ATCC | | 25923 |
| *A.baumannii* | Jia’s lab | | / |
| *K.pneumoniae* | Jia’s lab | | / |
| **Recombinant DNA** | **Source** | | **Identifier** |
| Plasmid: pFuse-hIgG1-Fc | InvivoGen | | pFuse-hIgG1-Fc |
| **Chemicals and Proteins** | **Source** | **Identifier** | |
| Collagenase type I | Sigma-Aldrich | C1639 | |
| Dulbecco’s modified Eagle’s medium and Nutrient Mixture F-12 | Thermo Fisher Scientific | 11320-082 | |
| Fetal bovine serum | Thermo Fisher Scientific | 10091148 | |
| Penicillin/streptomycin | Thermo Fisher Scientific | | 15140-122 |
| Pentobarbital sodium salt | Merk | | 1063180500 |
| Gentamicin | MCE | | HY-A0276 |
| 0.05% Trypsin-EDTA | Thermo Fisher Scientific | | 25300062 |
| Lipofectamine® 3000 Transfection Reagent | Thermo Fisher Scientific | | L3000008 |
| Dulbecco’s Modified Eagle’s Medium | Thermo Fisher Scientific | | 11965-092 |
| dialysis membranes 7000D | Solarbio | | YA1082-5M |
| polyethylene glycol-2000 | Sangon Biotech | | A601785-0500 |
| Opti-MEM | Thermo Fisher Scientific | | 31985070 |
| Lipofectamine® RNAiMAX Transfection Reagent | Thermo Fisher Scientific | | 13778075 |
| TRIzol | Thermo Fisher Scientific | 10296010 | |
| Cell lysis buffer for Western and IP | Beyotime | | P0013J |
| Protein A/G-Agarose | Abmart | | A10001M |
| RIPA buffer | Beyotim | | P0013C |
| **Critical Commercial Assays** | **Source** | | **Identifier** |
| RNeasy Micro Kit | Qiagen | | 74004 |
| Fast Site-Directed Mutagenesis Kit | TIANGEN | KM101 | |
| FastQuant RT Kit (With gDNase) | TIANGEN | KR106-02 | |
| SuperReal PreMix Plus (SYBR Green) | TIANGEN | | FP205-02 |
| LEGEND MAX™ Mouse GM-CSF ELISA Kit | Biolegend | | 439807 |
| **Software and Algorithms** | **Source** | | **Identifier** |
| Feature Extraction software version10.7.1.1 | Agilent | / | |
| Genespring version13.1 | Agilent | | / |
| Gel imaging system Tanon 5500 | Tanon | | / |
| GraphPad prism 8 | GraphPad | | <http://www.graphpad.com>  RRID:SCR_002798 |
| FlowJo software version V10 | Tree Star | | / |
| Image J 180 | National Institutes of Health | | / |

Arabic superscript numerals in the source of mice or strains mean the sequence numbers of the references which used the corresponding mice or strains.

**Supplementary methods**

**Making *Cd69* knockout mice and *Erk1* knockout mice via CRISPR/Cas9 system**

Firstly, two sgRNAs-targeting the introns on both sides of the targeted region of *Cd69* or *Erk1* were respectively constructed and transcribed in vitro. Then Cas9 mRNA and sgRNA will be co-injected into zygotes. Thereafter, the zygotes were transferred into the oviduct of pseudopregnant ICR females at 0.5 dpc. And F0 mice was birthed after 19~21 days of transplantation, all the offspring of ICR females (F0 mice) were identified by PCR and sequencing of tail DNA. And positive F0 mice were genetyped by the methods. Finally, crossing F0 mice with C57BL/6J mouse to build up heterozygous mice.

**Flow Cytometry**

Antibody to human Fc is in Table S2. Isolated cells were incubated with corresponding fluorescent labelled antibody at 4°C for 30 minutes and then washed with wash buffer (PBS with 2%FBS). The cells were tested on a BD LSRFortessa (BD Immunocytometry Systems), and the data were analyzed with FlowJo V10 (Tree Star).

**Quantitative real-time PCR**

Total RNA in ASCs infected with PAO1 for 0, 2 and 4 hours was extracted using TRIzol (Thermo Fisher Scientific), and then 500 ng RNA was reverse-transcribed for cDNAs using FastQuant RT Kit (With gDNase, TIANGEN). qPCR system was constructed using SuperReal PreMix Plus (SYBR Green, TIANGEN). PCR conditions were 10 minutes at 95°C, 40 cycles of 15 seconds at 95°C, 30 seconds at 60°C. The amounts of transcript were normalized to those of *β-actin*. The primers used in this study are listed in Table S1.

**ELISA**

ASCs were seeded in 12-well dishes, grown overnight. The culture medium was replaced by DMEM/F12, then cells were infected with PAO1 or other bacteria for cell-culture supernatants collection. GM-CSF concentrations in lung tissue lysate supernatants or cell-culture supernatants were measured by LEGEND MAX™ Mouse GM-CSF ELISA Kit (Biolegend) according to the manufacturer's protocol.

**Western blot**

ASCs were prepared, and solubilized at ice-cold RIPA buffer (Beyotime, Shanghai, China) supplemented with PMSF. After centrifuge, the supernatant was boiled in 1 × SDS loading buffer. After separation by 10% SDS-PAGE, proteins were transferred in wet tank system onto polyvinylidene fluoride membranes. Blots were blocked with 5% skim milk for 1 hour, incubated with corresponding antibody overnight at 4℃ and incubated with the corresponding horseradish peroxidase-conjugated second antibody for 1 hour. To get chromogenic detection data, the blots were exposed to gel imaging system (Tanon 5500) after adding Thermo ECL substrate solution. For detection of CD69, SDS-PAGE was on a 12% gel.

**REFERENCES**

1. Xia B, Sun L, Fan X, et al. A new model of self-resolving leptospirosis in mice infected with a strain of. *Emerg Microbes Infect.* 2017;6(5):e36 LID - 10.1038/emi.2017.1016 [doi].

2. Yang JW, Mao B, Tao RJ, et al. Corticosteroids alleviate lipopolysaccharide-induced inflammation and lung injury via inhibiting NLRP3-inflammasome activation. *Journal of cellular and molecular medicine.* 2020;24(21):12716-12725.

3. Fan LC, Lin JL, Yang JW, et al. Macrolides protect against Pseudomonas aeruginosa infection via inhibition of. *Am J Physiol Lung Cell Mol Physiol.* 2017;313(4):L677-L686 LID - 610.1152/ajplung.00123.02017 [doi].

4. Bucior I, Abbott J Fau - Song Y, Song Y Fau - Matthay MA, Matthay Ma Fau - Engel JN, Engel JN. Sugar administration is an effective adjunctive therapy in the treatment of. *Am J Physiol Lung Cell Mol Physiol.* 2013;305(5):L352-363 LID - 310.1152/ajplung.00387.02012 [doi].
