# Supplementary figures and images for: CD69 mediates the protective role of adipose tissue‐derived mesenchymal stem cells against Pseudomonas aeruginosa pulmonary infection
Source: Clin Transl Med. 2021 Nov 4;11(11):e563. doi: 10.1002/ctm2.563 (PMC8567058; doi:10.1002/ctm2.563)

A

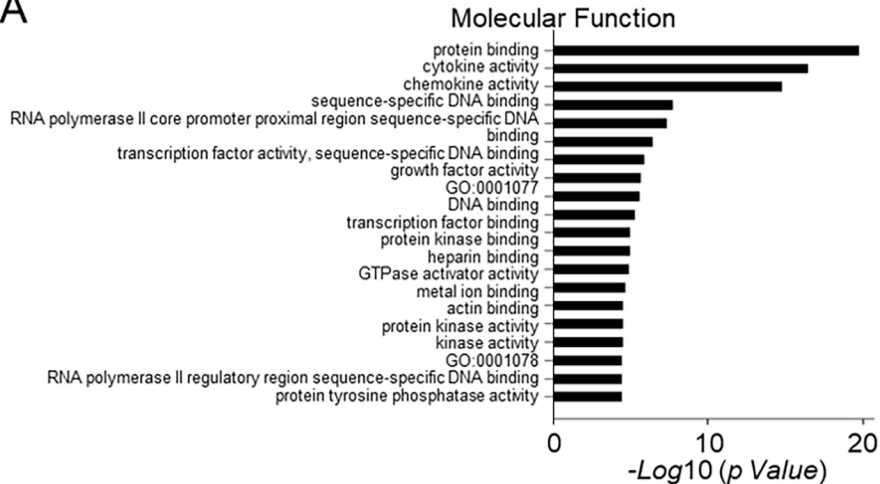

B

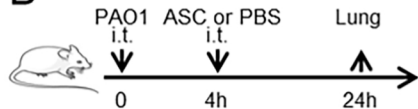

C

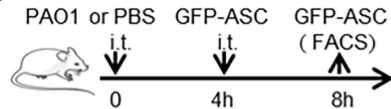

Supplement: Supplementary file 2 — Supporting information [file CTM2-11-e563-s006.pdf]

A

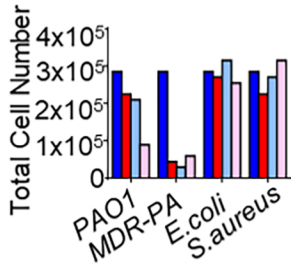

B

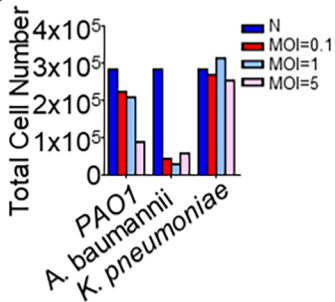

Supplement: Supplementary file 3 — Supporting information [file CTM2-11-e563-s004.pdf]

**A**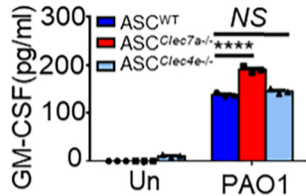**B**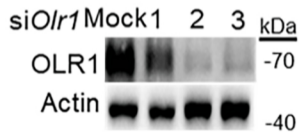**C**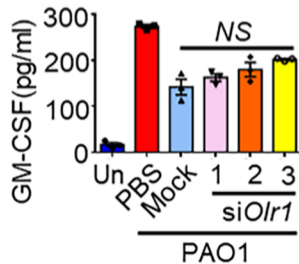**D**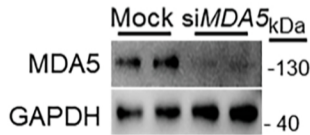**E**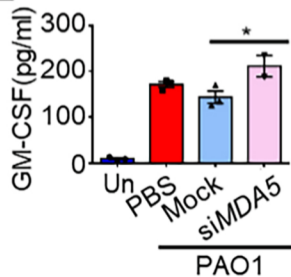

Supplement: Supplementary file 4 — Supporting information [file CTM2-11-e563-s002.pdf]

A

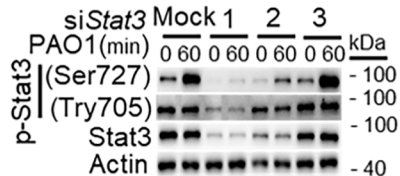

B

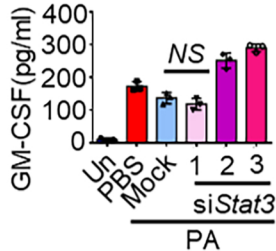

C

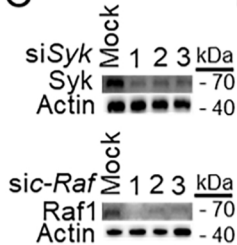

D

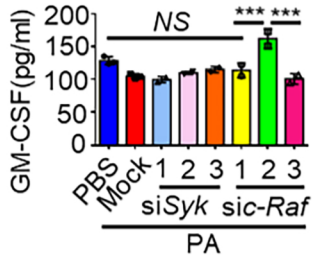

Supplement: Supplementary file 5 — Supporting information [file CTM2-11-e563-s001.pdf]

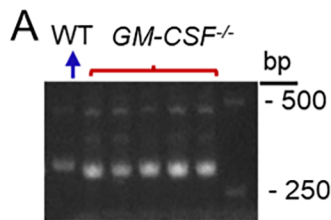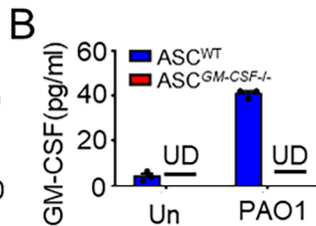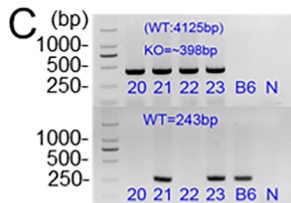

B6 (WT): Negative Control  
N: No-Template Control  
*Erk1*<sup>-/-</sup>: 20, 22  
*Erk1*<sup>+/-</sup>: 21, 23

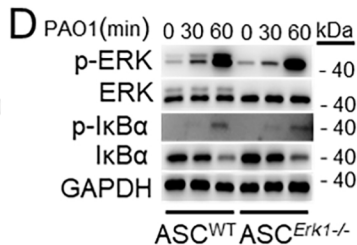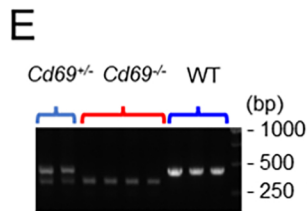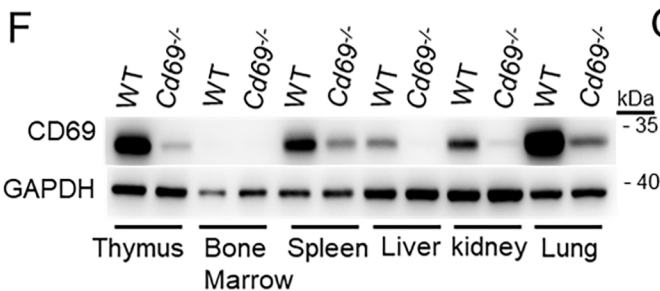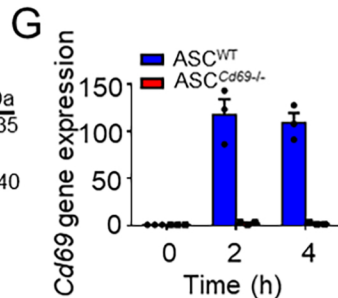

Supplement: Supplementary file 6 — Supporting information [file CTM2-11-e563-s003.pdf]

**A**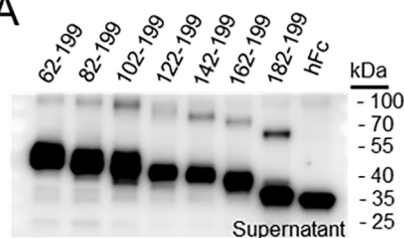**B**

The truncations of hCD69

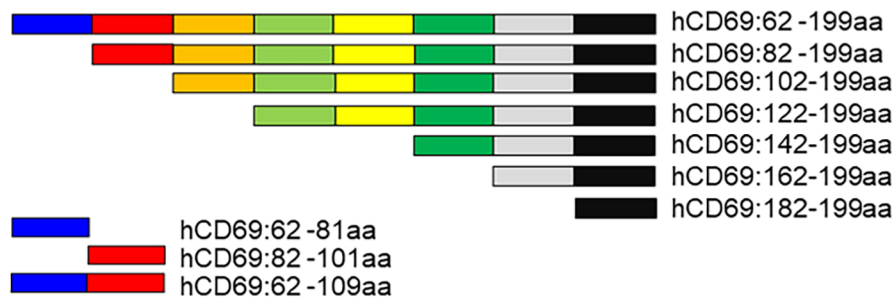**C**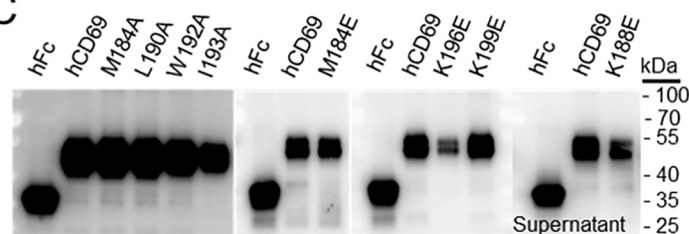**D**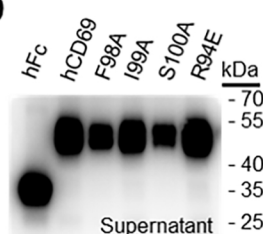**E**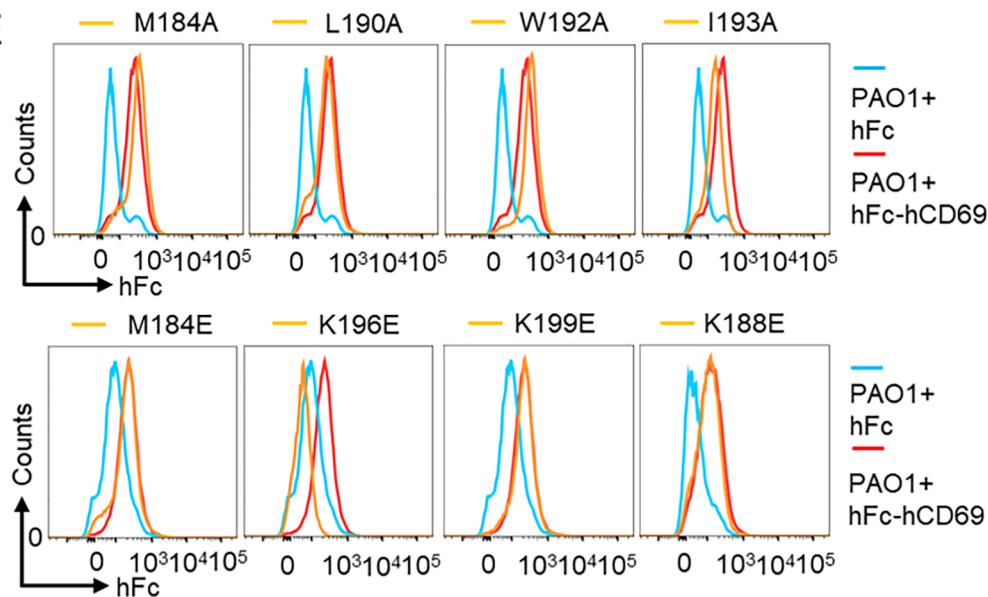

Supplement: Supplementary file 7 — Supporting information [file CTM2-11-e563-s007.pdf]
